# Investigating the metastability of amorphous calcium carbonate by droplet microfluidics experiments using machine learning

**DOI:** 10.1038/s41598-025-05984-0

**Published:** 2025-06-20

**Authors:** Ryan Santoso, Lisa Guignon, Guido Deissmann, Jenna Poonoosamy

**Affiliations:** 1 Institute of Fusion Energy and Nuclear Waste Management - Nuclear Waste Management (IFN-2), Forschungszentrum Jülich GmbH, 52428 Jülich, Germany; 2 Grenoble INP Ense3, Université Grenoble Alpes, 38000 Grenoble, France

**Keywords:** Droplet microfluidics, Machine learning, Amorphous calcium carbonate, Environmental chemistry, Optical spectroscopy, Computer science, Information technology, Scientific data, Lab-on-a-chip

## Abstract

Amorphous calcium carbonate (ACC) plays an important role in the crystallization pathways of calcite and its polymorphs influencing many natural and anthropogenic processes, such as carbon sequestration. Characterizing the dissolution rate of ACC in presence of additives of contaminants in favor of crystalline phases is challenging as such reactions occur readily in bulk solution. Droplet microfluidics offers a solution by confining ACC within a droplet, enabling a quantification of the transformation rate of ACC into crystalline phases. However, accurate quantification of this transformation requires analyzing more than thousands of droplets identifying the different polymorphs of calcium carbonate during an experiment, which is labor-intensive. Here we develop a visual-based machine learning method, combining cascading U-Net and K-Means clustering, to allow efficient analysis of droplet microfluidics experiment results. Using our method, we accurately inspect 11,288 droplets over 6 hours of experimental time to identify the polymorphs, using a CPU core in a laptop for only 42 minutes. This is achieved with manual labeling of 11 experimental microscopy images before augmentations. From our analyses the transformation rate of ACC into its crystalline phases can be inferred. The transformation rate indicates an increasing stability of the ACC phase in confinement. Our method is generalizable and can be applied to different setups of droplet microfluidics experiments, facilitating efficient experimentation and analysis of complex crystallization processes.

## Introduction

Calcium carbonate is ubiquitous in both natural and anthropogenic environments and is by far the most studied solid phase^1–5^. It occurs in various anhydrous crystalline phases: calcite (trigonal structure, space group R$\overline{3}$c), vaterite (hexagonal structure, space group $\text {P6}_{3}$/mmc), and aragonite (orthorhombic structure, space group Pmcn)^4,5^. Additionally, it forms hydrated phases such as monohydrocalcite (trigonal, space group $\text {P3}_{1}$12) and hexahydrocalcite (ikaite, monoclinic, space group C2/c), as well as amorphous (hydrous) calcium carbonate (ACC), with the latter being a potential precursor for the crystalline phases^4,5^. ACC can transform via dissolution and re-precipitation mechanisms, a pathway that often results in significant morphological changes in the final crystalline phase, or through solid-state transformation, which can preserve intricate hierarchical structures^1,2^. The transformation of ACC via dissolution and re-precipitation mechanisms is governed by equilibrium partition coefficients, maintaining the chemical composition of the aqueous solution. In contrast, during solid-state transformation, chemical redistribution can deviate significantly from equilibrium partitioning, preserving the original chemical composition of ACC in the crystalline end product^6^. Thus, each transformation pathway has a significantly different effect on element partitioning into the final crystalline phase^6^. Furthermore, the transformation of ACC to crystalline phases can result from a combination of solid-state transformation and dissolution-precipitation mechanisms^7^. Understanding calcium carbonate mineral formation from ACC precursors is highly relevant for various systems, not only for the biomineralization of marine calcifiers, which represents one of the long-term sinks of carbon on Earth^3,7^, but also for other environmental applications^8^. Indeed, the lack of selectivity with respect to trace element incorporation compared to crystalline calcium carbonate phases, along with the co-precipitation of trace elements via the formation of ACC precursors, make calcium carbonate formed via ACC a good candidate as a scavenger for various heavy metals^9^, including radionuclides^10,11^.

The fascinating transformation of ACC into complex shell and skeletal structures has triggered the exploration of bio-inspired crystal engineering approaches for controlling the transformation of ACC to its different crystalline phases as well as their morphology^4,5,12,13^. Given its critical importance, the characterization of ACC and the factors influencing its stabilization remains an active field of research^14^. Beside water^15–17^, impurities such as magnesium and phosphate are also known to affect the stability of ACC^18,19^. Other studies have shown that ACC is metastable in confinement even in the absence of additives^20–23^. The stabilization of ACC in confinement and in the presence of additives has significant implications for porous media research and subsurface remediation applications. For example, recent studies have shown that the addition of alkaline-earth metals, such as Ba, increases the stability of ACC in porous media, leading to unexpected precipitation patterns occurring in regions not characterized by the highest saturation ratio with respect to calcite^10^. Quantifying the metastability of ACC in the presence of additives and in confinement is crucial for understanding the transformation rate of ACC to crystalline phases, which in turn provides valuable insights for tailoring calcium carbonate phases for biomedical, bioengineering, and environmental remediation applications^5,24^. The determination of the transformation rate of ACC as a measure of its metastability can be used to quantitatively compare the effects of various environmental conditions, such as confinement, temperature, gas fugacities, or the presence/absence of other ions/additives, on its transformation^25^.

Microfluidics-based screening and lab-on-chip have emerged as powerful tools to investigate mineral dissolution and crystallization processes^26–28^, including minerals with highly radioactive elements e.g. radium-bearing barite^29^. These techniques can include in-situ spectroscopic techniques, such as Raman spectroscopy^30–32^ or synchrotron-based XRD^33,34^, to monitor phase transformations. Particularly, droplet microfluidics have been proposed as a high-throughput methodology to investigate ACC transformation^22,23^. This technique involves the formation of a series of aqueous droplets in a continuous phase (oil or air), with each droplet serving as an individual micro-batch experiment. Droplet microfluidics has been successfully applied to determine nucleation rates of various minerals^34,35^, including calcium carbonate^21^, and the transformation rate of ACC^25^. However, a significant challenge in deploying droplet microfluidics is the analysis of experimental results, which typically requires labor-intensive image segmentation to identify multiple crystals within droplets and track changes over time^35,36^. Here, we propose the use of visual-based machine learning methods to assist in this analysis.

In the context of droplet microfluidics, visual-based machine learning methods, such as Convolutional Neural Networks (CNN) and ResNet, have often been employed to optimize droplet generation in real time^37–41^. However, there has been less focus on using machine learning methods for evaluating experimental results, where analyzing the content of numerous droplets is required. This is particularly important for conducting multiple experiments under varying conditions to assess their impact on ACC transformation. A recent study uses a CNN model, trained and validated with a library containing 92,000 images, to identify bacterial growth in 3,000 droplets in an 20-minute experiment^42^. Similarly, another CNN model YOLOv5 is trained and validated with 643 droplet images and 2,063 cancer-cell images to identify PC3 cancer cells in 20 droplets per image^43^. These approaches, however, require a large number of manually labeled samples for the machine learning training.

In this study, we combine multiple U-Nets, namely the cascading U-Net method, the K-Means clustering method, and our identification algorithm (see Algorithm 2) to assist with analysis. The cascading U-Net method facilitates fast image segmentation, enabling accurate identification of droplets and multiple crystals over time with a small number of training samples required for training the machine learning models. To our knowledge, this is the first application of the cascading U-Net method for the fast identification of multiple crystals in droplet microfluidics experiments. The K-Means clustering method quantifies changes in minerals and droplets by analyzing the clustered colors in the segmented images produced by U-Net. Meanwhile, we develop an algorithm for tracking droplets containing ACC over time to infer its transformation rate. This approach is anticipated to reduce the laborious effort required for analyzing droplet microfluidic experimental results, thereby providing a toolbox for experimentalists to rationalize multiple experiments under different conditions.

## Methods

### Experimental setup

**Fig. 1:**
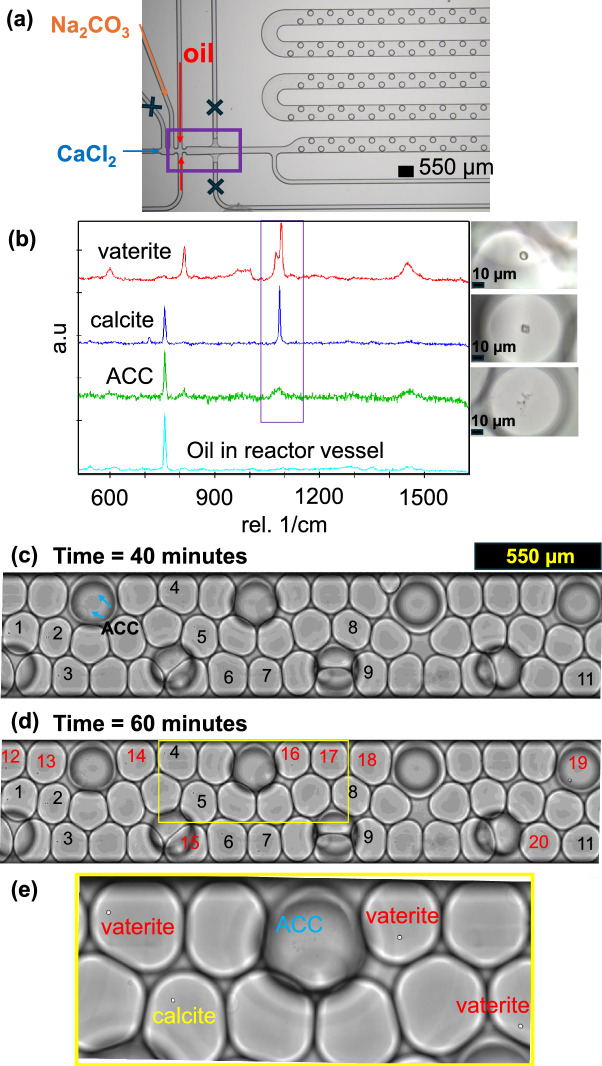
Experimental setup, Raman spectra, and snapshots of experimental result. (**a**) A detailed view of the inlet side consisting of two inlets (blue arrow: $\text {CaCl}_{2}$ and orange arrow: $\text {Na}_{2}\text {CO}_{3}$) dedicated for injection of aqueous solutions and two channels (red arrows) for oil circulation. The purple box shows the location where monitoring was performed to ensure well mixing the reactants at the nozzle. Several droplet traps are installed on the storage platform to trap the droplets during the monitoring process. The unlabelled channels are not used during the experiments, denoted by X marks. (**b**) Raman spectra confirming the three different phases and their associated morphologies, with the spectrum of the oil in the reactor shown in cyan. (**c**) Snapshot of the droplet storage channel after 40 minutes, with droplets numbered 1-11 indicating presence of crystalline phases. (**d**) Snapshot of the droplet storage channel after 360 minutes with additional droplets numbered 12-20 on top of the ones in (**c**) where ACC has transformed into crystalline phases. (**e**) Zoomed-in view of the yellow box ($800\,\upmu$m width) from (**d**), providing better visualization of the calcium carbonate phases: ACC phase (irregular shape), vaterite crystal (spherical shape), and calcite crystal (rhombohedral shape).

The droplet-microfluidic experimental setup follows that of our previous study^35^ and consists of a microfluidic reactor, an inverted microscope Eclipse Ti2 (NIKON, Tokyo) with a CFI Plan Fluor DL $\times$10 objective (numerical aperture 0.3; working distance 16 mm, Nikon, Tokyo), a high-resolution camera from Zyla (sCMOS, Andor, Belfast), and a pressure pump with flow controller (Fluigent, Jena). The microfluidic reactor is a commercial droplet generator chip with an integrated storage platform made out of TOPAS^®^ COC (cyclic olefin copolymer; Fluidic 719 from Microfluidic ChipShop, Jena) (see Figure 1a). A commercial chip is chosen over an in-house design for our experiments and analysis to make our developed visual-based, data-driven machine learning method more accessible to the geochemistry community. Two inlets are dedicated for the injection of the oil phase (Novec^TM^ 7500 fluorinated oil, Fluigent Le Kremlin-Bicetre) and two for the injection of the dispersed (aqueous) phase, i.e., a 10 mM calcium chloride ($\text {CaCl}_{2}$) solution and a solution of 10 mM of sodium carbonate ($\text {Na}_{2}\text {CO}_{3}$) to trigger the precipitation of $\text {CaCO}_{3}$ (see Figure 1a). The flow rates used for the experiment are 1 $\mu$L $\text {min}^{-1}$ for both $\text {CaCl}_{2}$ and $\text {Na}_{2}\text {CO}_{3}$ solutions and 20 $\mu$L $\text {min}^{-1}$ for the oil phase, resulting in droplets of about 170 $\mu$m diameter and c. 2 nL volume (calculated using the formulation in dos Santos et al. (2018)^44^). The storage platform includes droplet traps.

The experiment was conducted at room temperature ($22\,^{\circ }$C) which was continuously monitored throughout the experimental duration. The experimental setup was housed within an enclosed cabinet designed to minimize fluctuations in temperature, mechanical vibrations, and exposure to cosmic rays^35^. Under these conditions, no observable shrinkage in droplet volume occurred, indicating that the droplets remained stable throughout the duration of the experiments. The solution viscosity is 1 $\times \,10^{-3}$ Pa$\cdot$s.

The fabrication of droplets was enabled for 20 minutes and carefully monitored by the experimentalist, ensuring well mixing of the reactants at the nozzle (see purple box in Figure 1a) and consistent droplet generation, using the live camera of the microscope. After 30 minutes, the mineralogical transformations were monitored using time-lapse microscopy for 6 hours. Raman spectra, using a detailed protocol described in our previous study^10^ were collected to determine the polymorphism of the newly formed precipitates, see Figure 1b. The Raman spectroscopy was performed probing precipitates inside the droplets (at least 10 spectra collected for each morphology) using a Witec alpha300 Ri inverted confocal microscope with a Nikon CF plan 100$\times$ objective of numerical aperture 0.95. The instrument is equipped with a Nd:YAG laser ($\lambda$ = 532 nm), a thermoelectrically cooled CCD Camera, and a Ultra-High-Throughput Spectrometer UHTS300. Raman spot measurements were collected using a grating of 1800 grooves per mm at a laser power of 20 mW collecting 100 measurements each lasting 0.5 s for amorphous phases and 50 measurements each lasting 0.5 s for the crystalline phases. These measurement were collected after 24 hours.

In our analysis, we define $t_{0} =$ 30 minutes which denotes the start of the monitoring period using time-lapse microscopy. We further provide Supplementary Information 1 containing snapshots captured at $t_{0}$, demonstrating well mixing condition and absence of vacant droplets. For higher resolution snapshots, we encourage readers to access our data repository https://doi.org/10.26165/JUELICH-DATA/RZ8RZ3.

### Linking Raman spectroscopy and crystal morphology

In our experiment, three distinct phases - amorphous calcium carbonate, vaterite, and calcite - were visually identified (see Figure 1c to e) and further confirmed using Raman spectroscopy (see Figure 1b). The calcite is characterized by the $v_{4}$ in-plane bending mode of $\text {CO}_{3}^{2-}$ at 712 $\text {cm}^{-1}$, the $v_{1}$ symmetric stretching mode of $\text {CO}_{3}^{2-}$ at 1085 $\text {cm}^{-1}$, and the $v_{3}$ antisymmetric stretching mode of $\text {CO}_{3}^{2-}$ at 1436 $\text {cm}^{-1}$^45^. Morphologically, calcite is identified by its characteristic rhombohedral shape^46^. The vaterite can be distinguished from calcite by the doubling of the $v_{1}$ band, which produces peaks at 1074 and 1090 $\text {cm}^{-1}$^45^. It is visually characterized by a spherical shape^23^. The ACC phase, on the other hand, is readily distinguished by the absence of a sharp $v_{1}$ band, exhibiting instead a broader and less intense band^45^. This phase is visually identified by its irregular morphology^47^. This link between Raman spectral signatures and the visual morphologies of the solid phases enables the application of visual-based machine learning methods to further analyze our experimental results. Note that given the high concentration of reactants used in our experiment, ACC is instantaneously formed in all droplets upon fabrication.

### Cascading U-Net and K-Means clustering method

We use a visual-based, data-driven machine learning method to enable fast analysis of droplet microfluidic experiment results, distinguishing between droplets, vaterite, and calcite. Our strategy for inferring the number of droplets containing the ACC phase is based on counting the number of droplets containing either vaterite or calcite crystals, as all droplets initially contain the ACC phase (see Supplementary Information 1). Additionally, ACC has lower color contrast (see the ACC phase in Figure 1e), making it challenging for image segmentation.

**Fig. 2:**
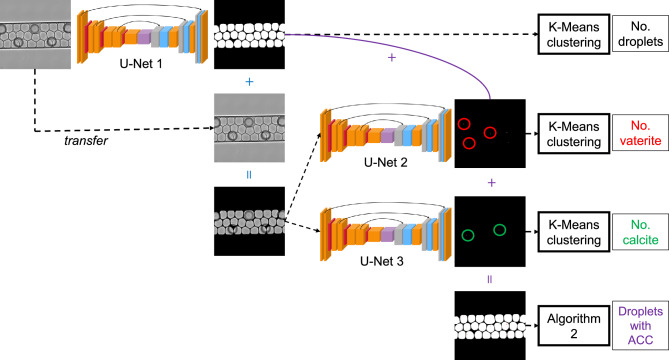
Cascading U-Net approach consisting of three U-Nets dedicated for droplet, vaterite, and calcite segmentation. The red circles denote vaterite pixels, and the green circles denote calcite pixels. ”+” symbol denotes stacking operations, described in Algorithm 1.

We rely on the U-Net^48^ as the backbone of our approach for segmentation. Three U-Nets, as shown in Figure 2, are employed to accurately identify the droplets, vaterite, and calcite crystals, respectively. This three-U-Net approach, namely cascading U-Net method, addresses the issue of feature imbalance, where vaterite and calcite- our primary segmentation targets-occupy a maximum of only three pixels, whereas droplets cover significantly more pixels (see Figure 1c). Additionally, there are high-contrast backgrounds that must be handled during segmentation. These conditions can obscure the vaterite and calcite crystals, leading to a trained model biased toward the dominant feature (i.e. the droplets). As a result, accurately distinguishing vaterite and calcite crystals becomes challenging without a large number of training samples^49–51^. However, generating such a large number of training samples would be labor-intensive. Our proposed method reduces the need for an extensive number of training samples while still providing accurate predictions.

The approach begins with the first U-Net model, which is used to segment RGB experimental snapshots obtained through optical microscopy, specifically for identifying the droplets (see the output of the first U-Net model in Figure 2). This model takes RGB images as inputs and produces binary images, where droplets are represented in white and the background in black.

After obtaining the optimally trained first U-Net model, the next step is to overlay the predictions onto the input images, as illustrated by the blue ”+” sign in Figure 2. This overlay process follows Algorithm 1. The purpose of this step is to remove contrast from the background, allowing the subsequent segmentation to focus solely on the features within the droplets. Without this step, the issue of feature imbalance would persist.

**Algorithm 1:**
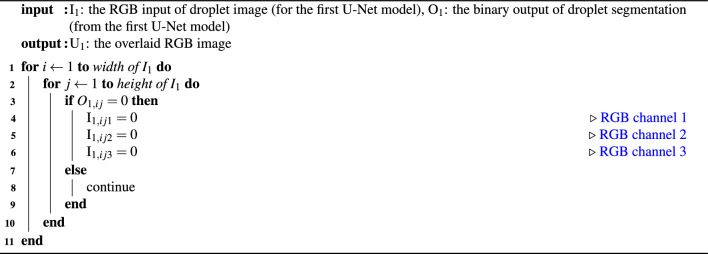
Algorithm for overlaying operation.

The results of the overlay process are then used as inputs for the second and third U-Net models. Since these two models operate independently, they can be trained in parallel. The outputs of the second and third U-Net models are vaterite and calcite, respectively, represented as white pixels, with the droplets and background in black, as shown in Figure 2. The construction of the U-Net models and the generation of training samples are described in detail in Supplementary Information 2.

After training all U-Net models, we can now obtain binary outputs for identifying droplets, vaterite, and calcite. These outputs are then fed into the K-Means clustering algorithm to determine the number of droplets, and vaterite and calcite crystals. In this setup, the calculation of clusters is straightforward, as there are clearly two clusters, white-pixel and black-pixel clusters. We use the centers of the white-pixel cluster to calculate the number of droplets and crystals.

By stacking the binary outputs of droplet, vaterite, and calcite segmentation (overlay operation shown in Figure 2 with purple color), we achieve a complete segmentation of the original input images, as shown in Figure 2, with the background in black, droplets in white, vaterite in red, and calcite in green. These stacked images are useful for determining the transformation rate of ACC using Algorithm 2. The main purpose of Algorithm 2 is to calculate the number of droplets over time that contain ACC. This algorithm relies on a finding-contours and minimum-enclosing-circle algorithm provided by the OpenCV Python library^52^.

**Algorithm 2:**
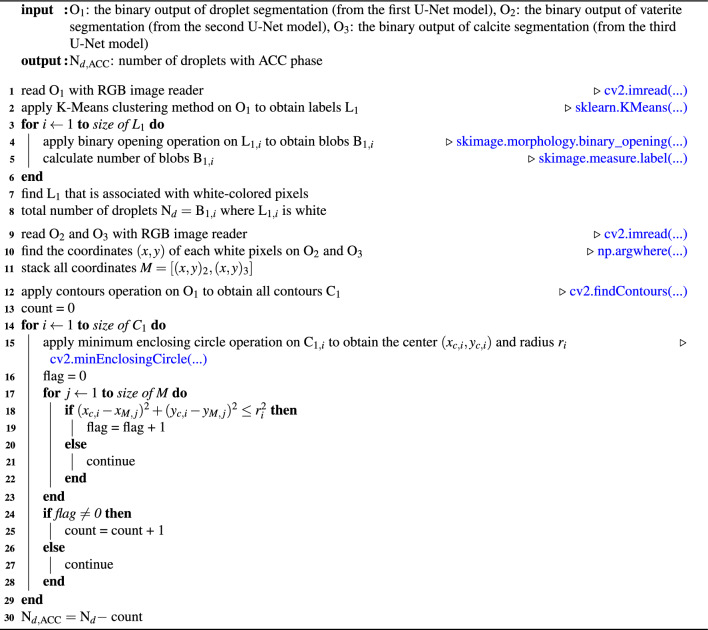
Algorithm for detecting droplets with ACC phase. This algorithm uses four Python libraries: OpenCV^52^ represented as cv2, scikit-learn^53^ represented as sklearn, scikit-image^54^ represented as skimage, and numpy^55^ represented as np.

The cascading U-Net combined with the K-Means clustering method is general and can be extended to other problems. To apply this approach to different problems, the first step is to identify the dominant features that might obscure the minor features of interest. If multiple dominant features are present, the first U-Net model can be expanded into several U-Net models. The overlay operation then removes the dominant features before proceeding to segment the minor features. The number of U-Net models for minor feature segmentation can also be adjusted based on the number of minor features present among the dominant features. Once all U-Net models are trained, the K-Means clustering method can be applied to the output of each model to facilitate quantitative analysis. All implementations described here are within our open-source Python library, Chip Analyzer and Calculator (cac). The library can be downloaded from our Github repository https://github.com/FZJ-RT/cac.git.

An important consideration is the resolution of the input images to the U-Net models. The images must provide sufficient clarity of both crystal and droplet features, preserve spatial information, and balance the computational cost of model training. A cut-and-resize approach (see Supplementary Information 2) is recommended for handling high-resolution optical images (e.g. 7975$\times$16381$\times$3 pixels in our case study). However, we advise limiting the number of cuts to a maximum of two to reduce artifacts during the reconstruction process after segmentation. The resizing dimensions should be then chosen to maintain adequate feature clarity while considering the computational efficiency of the U-Net models training.

## Results

### Cascading U-Net results

We conducted a droplet microfluidics experiment over 6 hours, capturing multiple snapshots from 16 different locations (each covering an area of 3 mm $\times$ 1.5 mm) at 17 time steps per location, with each snapshot containing approximately 50 droplets. For generating training samples, we used snapshots from 4 different locations. At one location, we manually labeled snapshots from 5 time steps, representing half of the experimental period. For the other three locations, we labeled only 2 time step snapshots each. The locations and time steps are randomly selected. We then split the data into training and validation sets, with an 80:20 ratio. We also ensure that the selected 4 locations and their corresponding 17 time steps are excluded from the prediction samples.

**Fig. 3:**
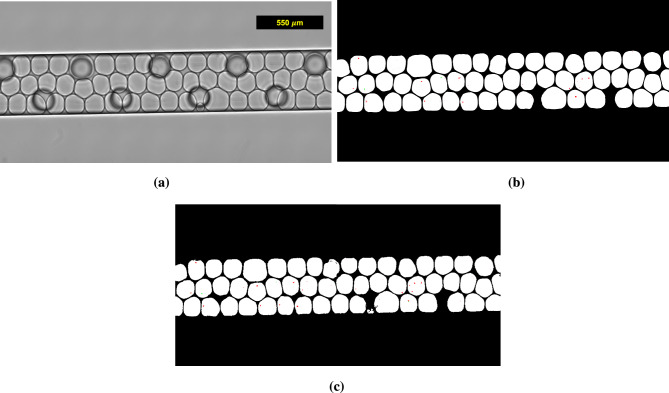
An exemplary segmentation result using our cascading U-Net method. It contains 54 droplets out of 11,288 droplets (in total): (**a**) the experimental snapshot at time = 22.72 minutes, (**b**) the ground truth, and (**c**) the prediction result. The presented images (**a**) to (**c**) are combined images after going through the cutting process in Figure S2.1 in Supplementary Information 2. The green pixels indicate calcite crystals, red pixels indicate vaterite crystals, and bubbles without both red and green pixels are classified as containing the ACC phase.

Figure 3 shows the prediction result at time = 22.72 minutes, combining two prediction outputs from the cascading U-Net method. The first U-Net model successfully captures most of the droplet boundaries. This accurate segmentation for each cut image results in a smooth and well-merged final image. While the second U-Net model slightly overpredicts the number of vaterite crystals, the third U-Net model accurately identifies the location and quantity of calcite crystals. For the remaining 191 optical microscopy images and for high resolution images, we encourage readers to visit our data repository https://doi.org/10.26165/JUELICH-DATA/RZ8RZ3. Based on these results, we are confident in using our trained U-Net models for further analysis of the transformation rate of ACC.

Table 1 outlines the performance of each U-Net model used in the cascading U-Net method. All three U-Net models exhibit similar offline and online processing times, reflecting their comparable architectures and hyperparameter setups. An offline time refers to the time needed for constructing a machine learning model and optimizing its hyperparameters, while an online time for deploying trained machine learning models for prediction. The variation in error magnitude is due to the different amounts of features (white pixels) each model must segment. For example, the first U-Net model has a larger error than the second because it segments more white droplet pixels compared to the vaterite pixels. However, these errors are acceptable, as demonstrated by similarity of the prediction result shown in Figure 3c and the ground truth in Figure  3b. All U-Net models are trained using a GPU in the JURECA High-Performance Computing (HPC) infrastructure at Forschungszentrum Jülich, utilizing a GPU-equipped node with 2 $\times$ AMD EPYC 7742 processors (2 $\times$ 64 CPU cores at 2.25 GHz), 512 GB of RAM, and 4 $\times$ NVIDIA A100 GPUs per node. The online phase is computationally cheap, running on a CPU in a Dell Latitude 7440 Laptop equipped with Gen 13th Intel i7-1365U containing 12 CPU cores at 1.8 GHz and 32 GB RAM. We can produce the result in Figure 3c in approximately 7 seconds. Using this method, we can analyze 11,288 droplets (in total), along with vaterite and calcite identification, across 192 optical microscopy images in 42 minutes.

**Table 1:** Performance of each U-Net model within the cascading U-Net method. For offline phase, we use a GPU in JURECA High-Performance Computing (HPC) infrastructure at Forschungszentrum Jülich, utilizing a GPU-equipped node with 2 $\times$ AMD EPYC 7742 processors (2 $\times$ 64 CPU cores at 2.25 GHz), 512 GB of RAM, and 4 $\times$ NVIDIA A100 GPUs per node. For online phase, we employ a CPU in a Dell Latitude 7440 Laptop equipped with Gen 13th Intel i7-1365U containing 12 CPU cores at 1.8 GHz and 32 GB RAM.

Model	Number of encoders	Number of filters in the first encoder	Learning rate	Batch size	Number of epochs	Offline time (minutes)	Offline resources	Binary cross-entropy error	Online time per image (seconds)
U-Net 1	5	32	$4.4 \times 10^{-4}$	2	9,269	15	1 GPU	$2.7 \times 10^{4}$	0.46
U-Net 2	6	64	$4.9 \times 10^{-4}$	16	3,853	17	1 GPU	$7.7 \times 10^{2}$	1.60
U-Net 3	5	64	$6.9 \times 10^{-4}$	13	2,997	16	1 GPU	$2.1 \times 10^{2}$	1.22

### Clustering and counting results

Table 2 presents the performance of each K-Means clustering method. The offline phase is relatively short because, unlike the U-Net models, the K-Means method does not involve as many weights and parameters to optimize. The online phase is also quick, enabling fast analysis to estimate the transformation rate of ACC.

**Table 2:** Performance of each K-Means clustering method. K-Means 1 refers to the clustering for the outputs of the U-Net 1, K-Means 2 for the U-Net 2, and K-Means 3 for the U-Net 3. For both offline and online phase, we use a CPU in a Dell Latitude 7440 Laptop equipped with Gen 13th Intel i7-1365U containing 12 CPU cores at 1.8 GHz and 32 GB RAM.

Model	Offline time (seconds)	Offline resources	Online time per label (seconds)
K-Means 1	0.24	1 CPU	$7.3 \times 10^{-6}$
K-Means 2	0.18	1 CPU	$5.2 \times 10^{-6}$
K-Means 3	0.17	1 CPU	$6.7 \times 10^{-6}$

**Fig. 4:**
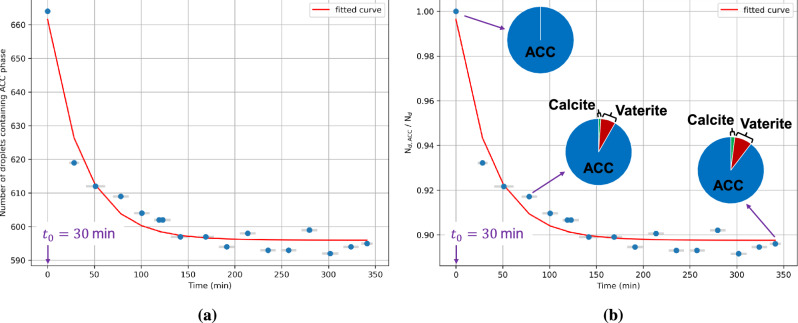
The analysis results of 11,288 droplets (in total) across 192 optical microscopy images. The gray bars represent the time discrepancies resulting from the sequential operation of taking the snapshots, while the blue dots indicate the total number of droplets containing the ACC phase, as analyzed from the cascading U-Net method outputs. The presented graphs are (**a**) the droplet count containing the ACC phase and (**b**) the ratio between then number of droplets containing the ACC phase and the initial number of droplets.

Figure 4 presents the analysis results using the K-Means clustering method and Algorithm 2. The blue dots at each time step represent the number of droplets containing the ACC phase, calculated across 12 locations (dedicated for the online phase). Since the snapshots of these 12 locations are taken sequentially, the time discrepancies are shown as bars, with the blue dots indicating the mean time. Overall, the data points follow an exponential decay trend. We fit the data with the following equation:

1 $$\begin{aligned} \text {N}_{d,\text {ACC}} = 65.63 \text {e}^{-0.027t} + 596.0 \end{aligned}$$

or for the ratio format:

2 $$\begin{aligned} \left( \frac{\text {N}_{d,\text {ACC}}}{\text {N}_{d}} \right) = 0.099 \text {e}^{-0.027t} + 0.898 \end{aligned}$$

where $\text {N}_{d,\text {ACC}}$ denotes the number of droplets containing ACC, $\text {N}_{d}$ denotes the total number of droplets, and *t* denotes time in minutes. The transformation rate obtained from fitting equation is 0.027 $\text {min}^{-1}$. The results show that approximately 90% of the total droplets still contain the ACC phase at the end of the experimental duration (6 hours). This suggests that ACC is stable for 6 hours experimental time.

To further confirm that the ACC remains stable over a long period in confinement, we take additional snapshots at 12 designated prediction locations after 24 hours and perform manual counting. Readers are encouraged to check it in Supplementary Information 3 or in our data repository in https://doi.org/10.26165/JUELICH-DATA/RZ8RZ3. We identify 653 droplets, a difference of 11 droplets compared to our proposed approach (this difference can be attributed to the error in the cascading U-Net method due to over prediction), with 443 droplets containing the ACC phase. This results in a ratio $\frac{\text {N}_{d,\text {ACC}}}{\text {N}_{d}}$ of 0.678. Therefore, the half-life is greater than 24 hours.

## Discussion

Using the droplet microfluidics setup, we are able to quantify ACC transformation rate in a confined volume of aqueous solution. As shown in Figure 4 and indicated by the manual counting results after 24 hours, a significant portion of the ACC phase remains stable throughout the experiment. This result aligns with previous studies which demonstrated similar stabilization of ACC^21^ and the preferential formation of the less stable crystalline phase, vaterite^22,23^. This stability is due to the confinement effect imposed by the droplet, placing the ACC phase in a metastable equilibrium with the solution inside the droplet^21^. For a comparison, without imposing any confinement, the lifetime of ACC phase is less than 20 minutes observed in micronized batch experiments^10^. The metastability of aqueous solutions is often observed in confinement^35^. For instance, no precipitation of barite occurs in droplet experiments, despite the solution being supersaturated with respect to barite^35^. An explanation for this metastability is the limited availability of aqueous ions required for the formation of crystals, reducing the driving force required to surpass the energy barrier for the formation of more stable phases^22^. This effect diminishes as the droplet size increases^35^. In the presented experiment, stabilization of the ACC phase is influenced by factors such as droplet size and the presence of additives of trace elements^21,25,56^, which can be further studied and quantified using the presented experimental setup using the commercial chip used here and accompanying analysis method. It should be noted that comparing experimental results using different chip designs may lead to varying outcomes, as the fluid injection rates, nozzle sizes, and droplet volumes are known to affect fluid mixing and, consequently, precipitation rates^22^. Therefore, for consistent comparison of the effects of additives, temperature, and other variables on ACC stability, the chip design should remain uniform across experiments.

The combined cascading U-Net and K-Means clustering method achieves accurate segmentation and identification results with a small number of manually labeled training samples. In contrast, the DeepLabV3+ model^57^, a widely used approach for multi-label segmentation, shows limitations with the same number of manually labeled samples (see Supplementary Information 4). The DeepLabV3+ model necessitates a significantly larger number of labeled training samples, which is less desirable due to the labor-intensive nature of manual labeling. Our method is also efficient, featuring a fast online phase and reasonable resource requirements for the offline phase. Moreover, accuracy can be further enhanced by incorporating additional manually labeled samples.

Our method is general and can be applied to various droplet microfluidic experimental setups. The key is to first separate the droplet features and then use multiple U-Net models to identify crystal features. In the future, we plan to extend our experiments by investigating the effect of stabilizing agents and different droplet sizes on the ACC transformation rate. These enhancements will also enable us to conduct more comprehensive analyses using the combined cascading U-Net and K-Means clustering method and further validate the effectiveness of the method.

## Supplementary Information

Supplementary Information 1.

Supplementary Information 2.

Supplementary Information 3.

Supplementary Information 4.

## Data Availability

The data for training is published on https://doi.org/10.26165/JUELICH-DATA/RZ8RZ3. The **Chip Analyzer and Calculator (cac)** Python library is in our Github repository https://github.com/FZJ-RT/cac.git. The **deeplab** Python library is also available in our Github repository https://github.com/FZJ-RT/deeplab.git.
