# Supplementary material for: Investigating the metastability of amorphous calcium carbonate by droplet microfluidics experiments using machine learning
Source: Sci Rep. 2025 Jun 20;15:20178. doi: 10.1038/s41598-025-05984-0 (PMC12181231; doi:10.1038/s41598-025-05984-0)
Supplement: Supplementary file 1 — Supplementary Information 1. [file 41598_2025_5984_MOESM1_ESM.pdf]

Supplementary Information 1 for:

Investigating the metastability of amorphous calcium carbonate by droplet microfluidics experiments using machine learning

Ryan Santoso[1], Lisa Guignon[1,2], Guido Deissmann[1], and Jenna Poonoosamy[1]

[1]Institute of Fusion Energy and Nuclear Waste Management – Nuclear Waste Management (IFN-2), Forschungszentrum Jülich GmbH, 52428 Jülich, Germany

[2]Grenoble INP Ense3, Université Grenoble Alpes, 38000 Grenoble, France

ABSTRACT

This supplementary information provides snapshots at t_0 showing well mixing condition and the absence of vacant droplets.

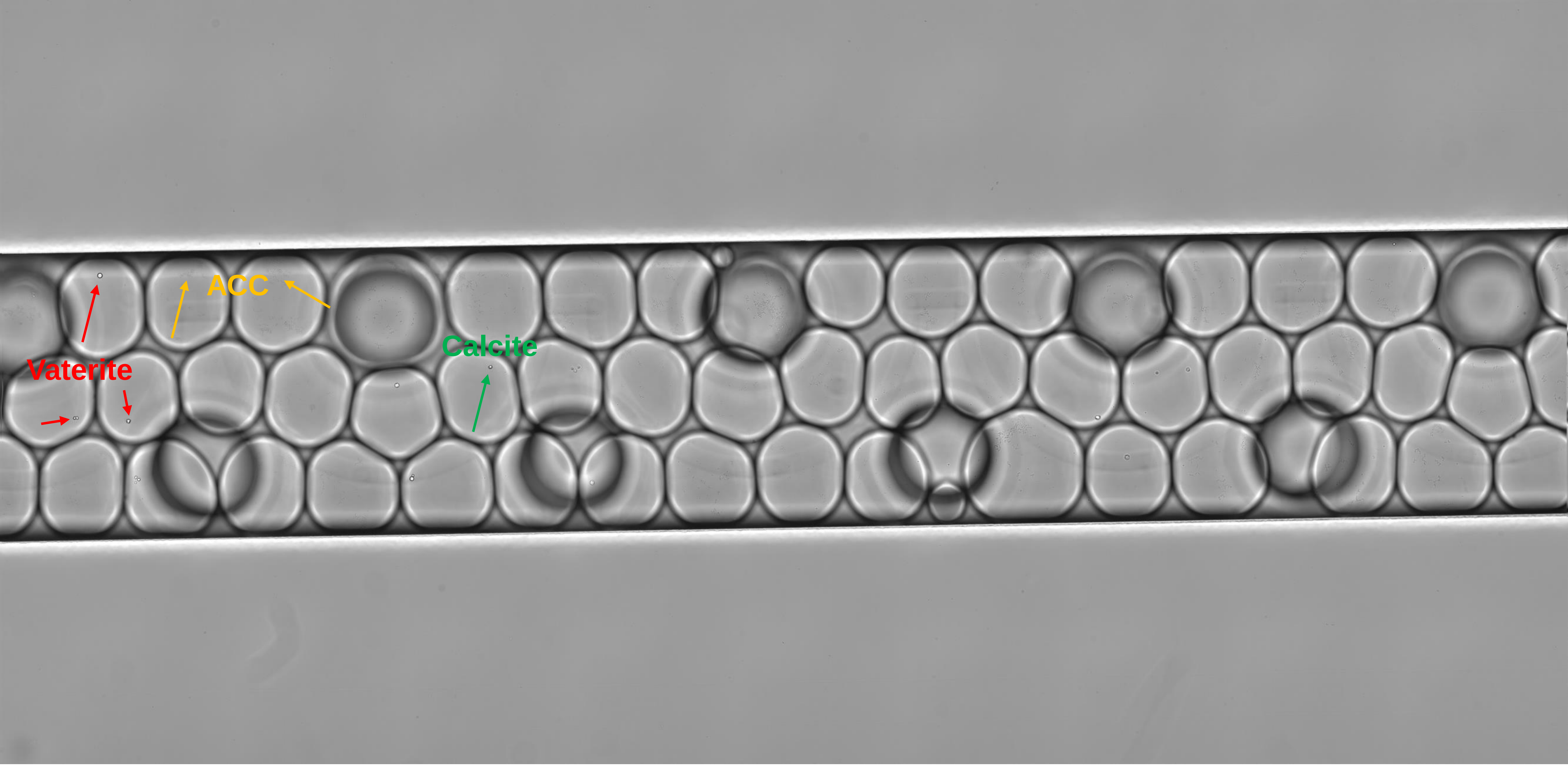

Location: 4

No droplets: 53

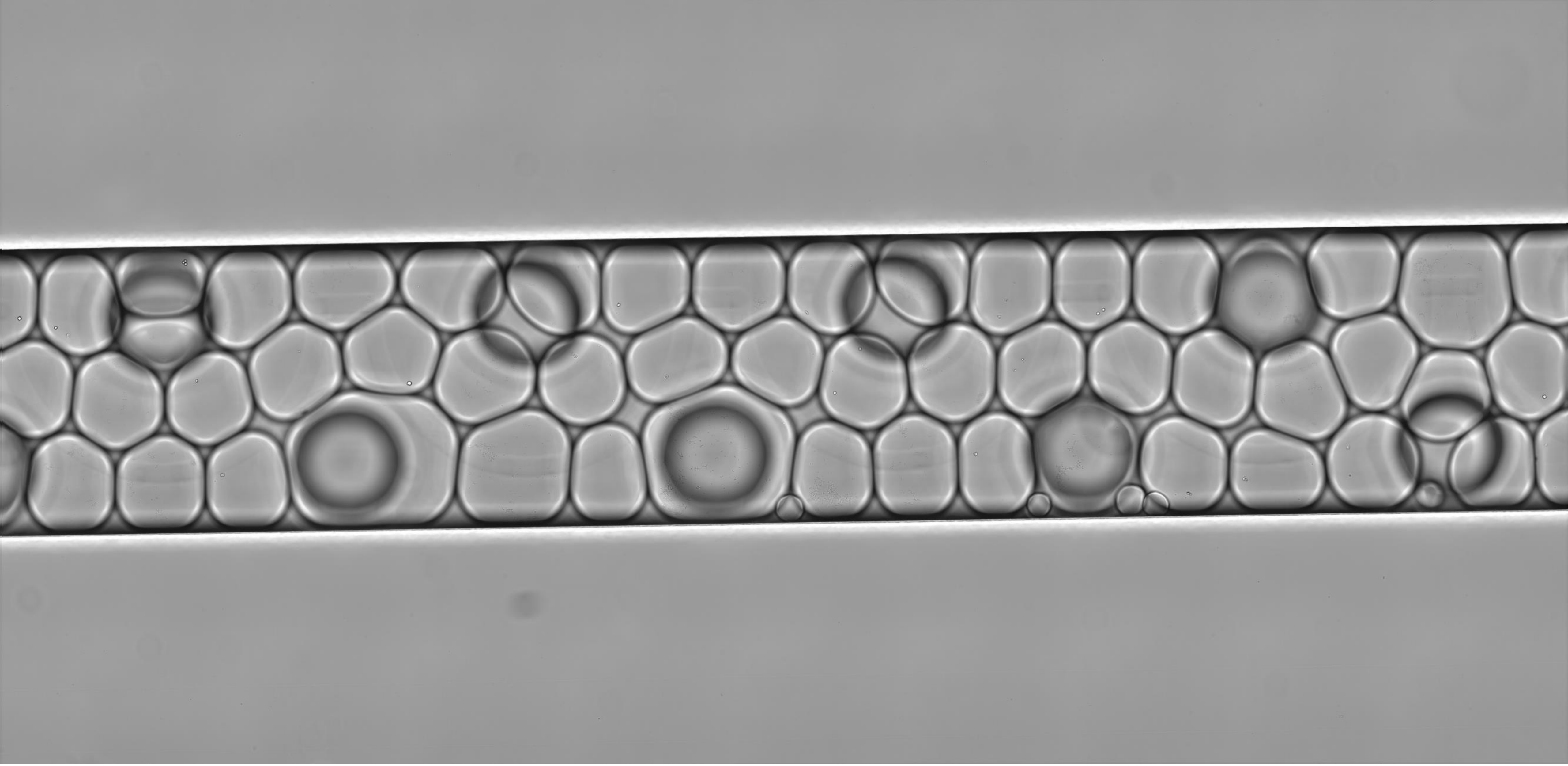

Location: 6 No droplets: 57

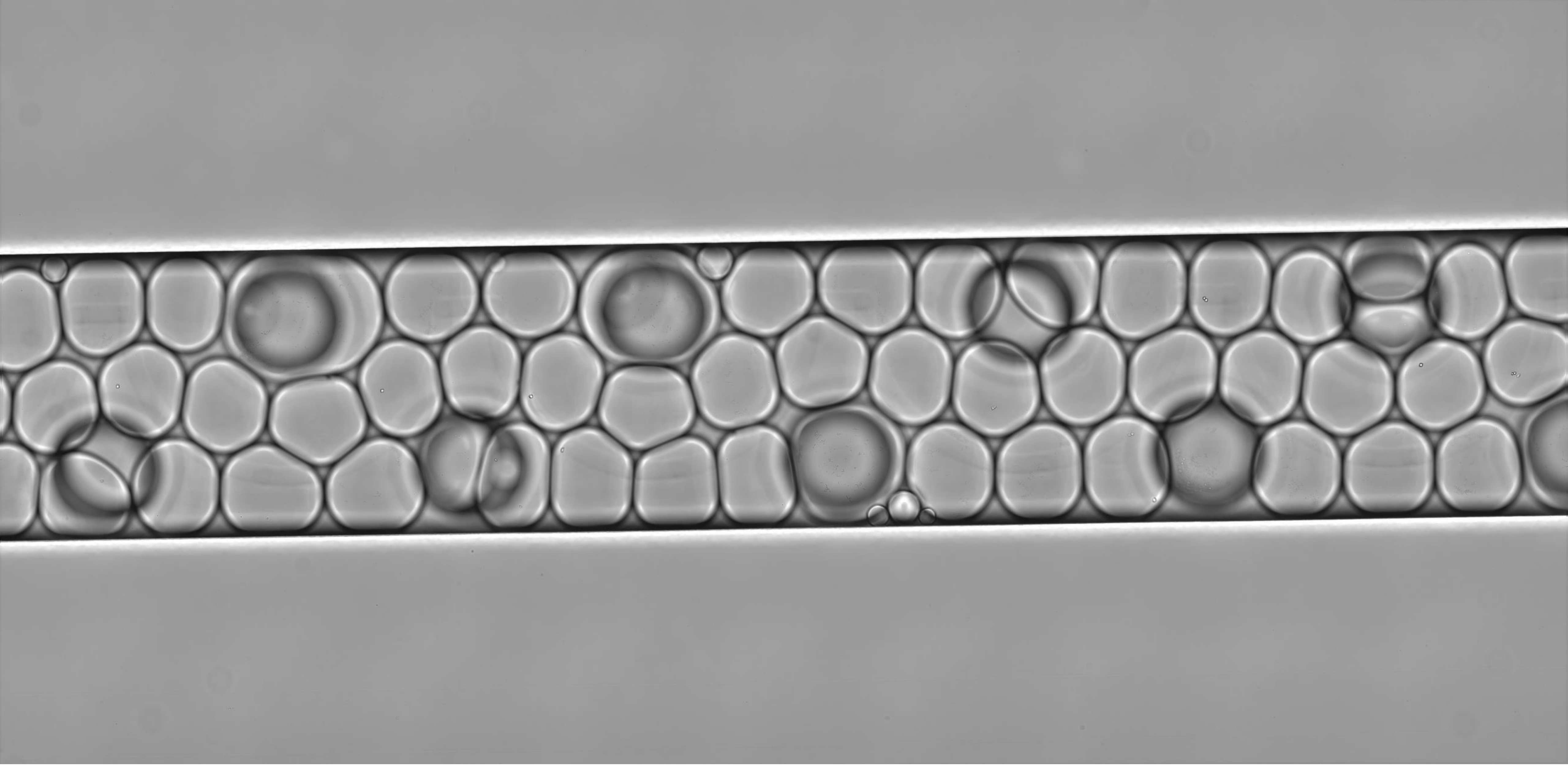

Location: 7 No droplets: 59

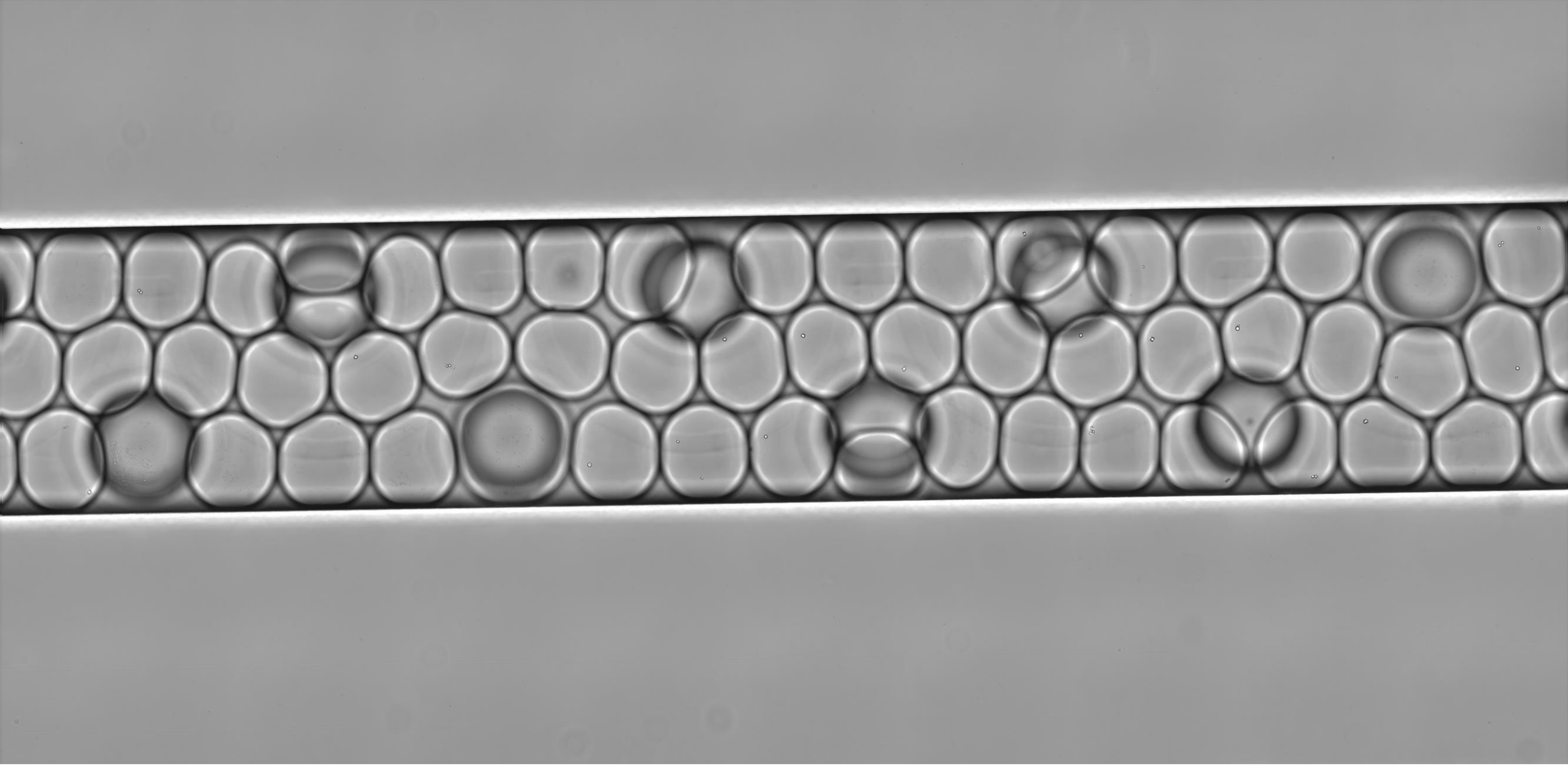

Location: 8   No droplets: 57

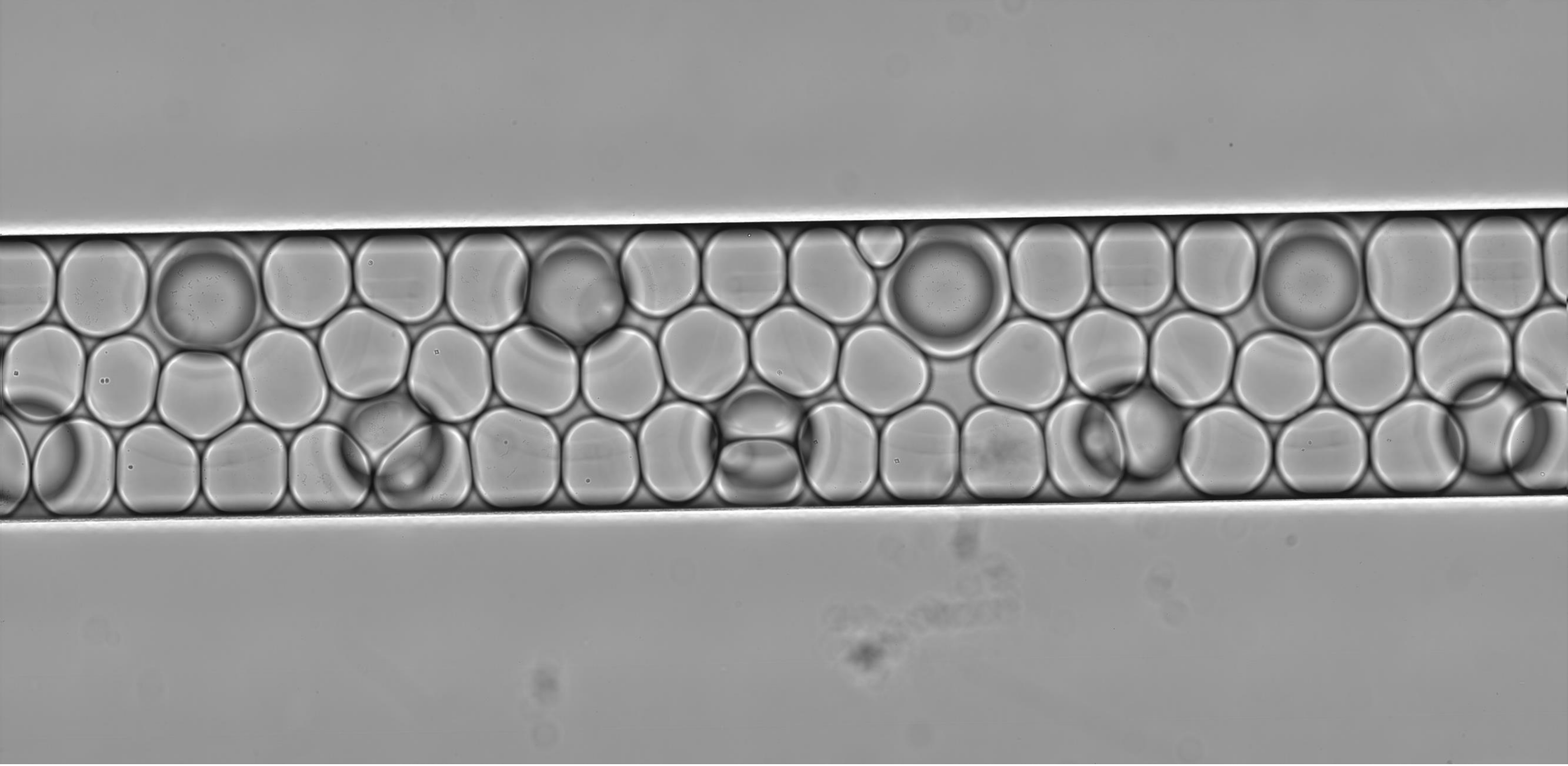

Location: 9 No droplets: 53

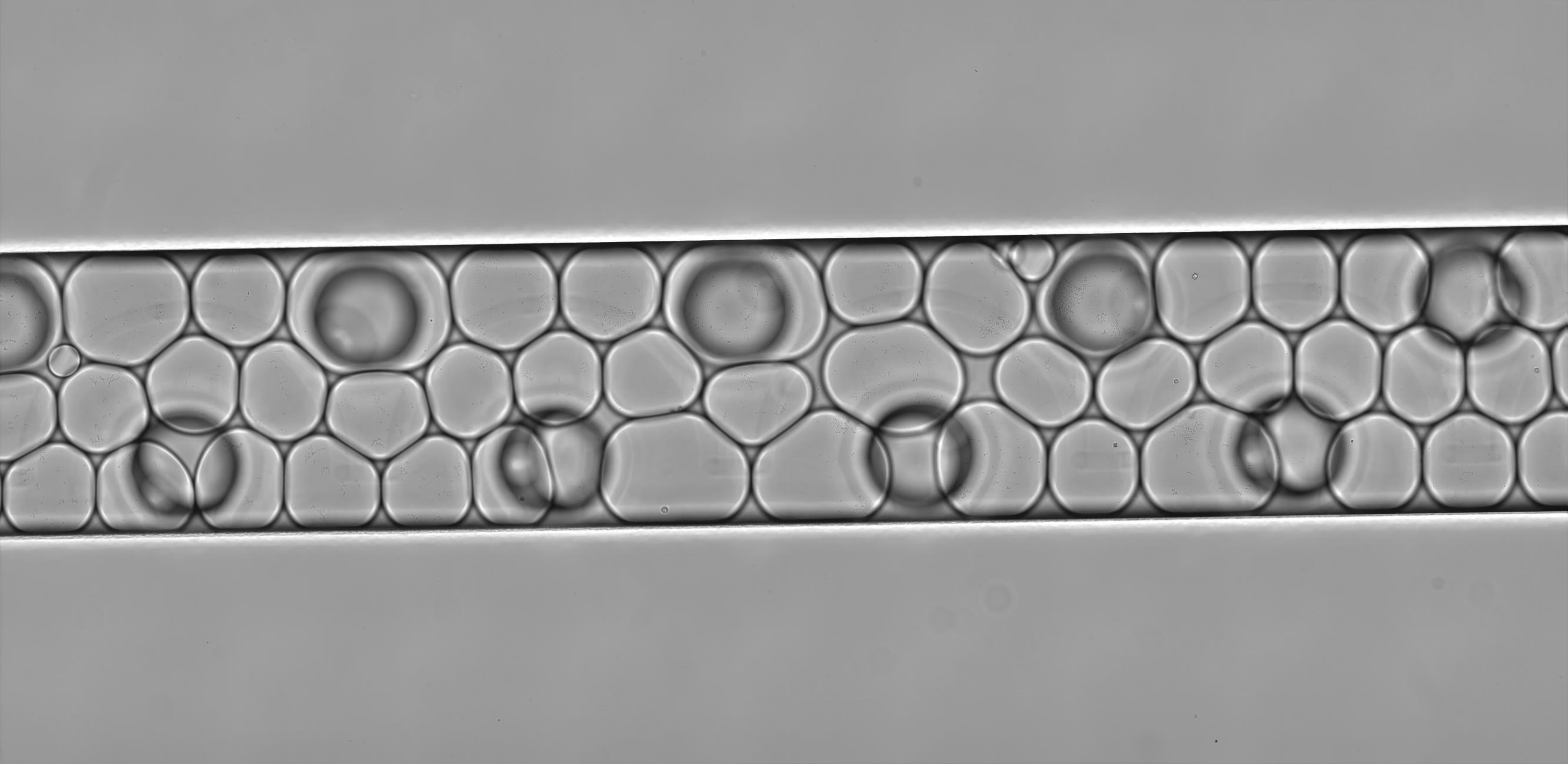

Location: 10 No droplets: 49

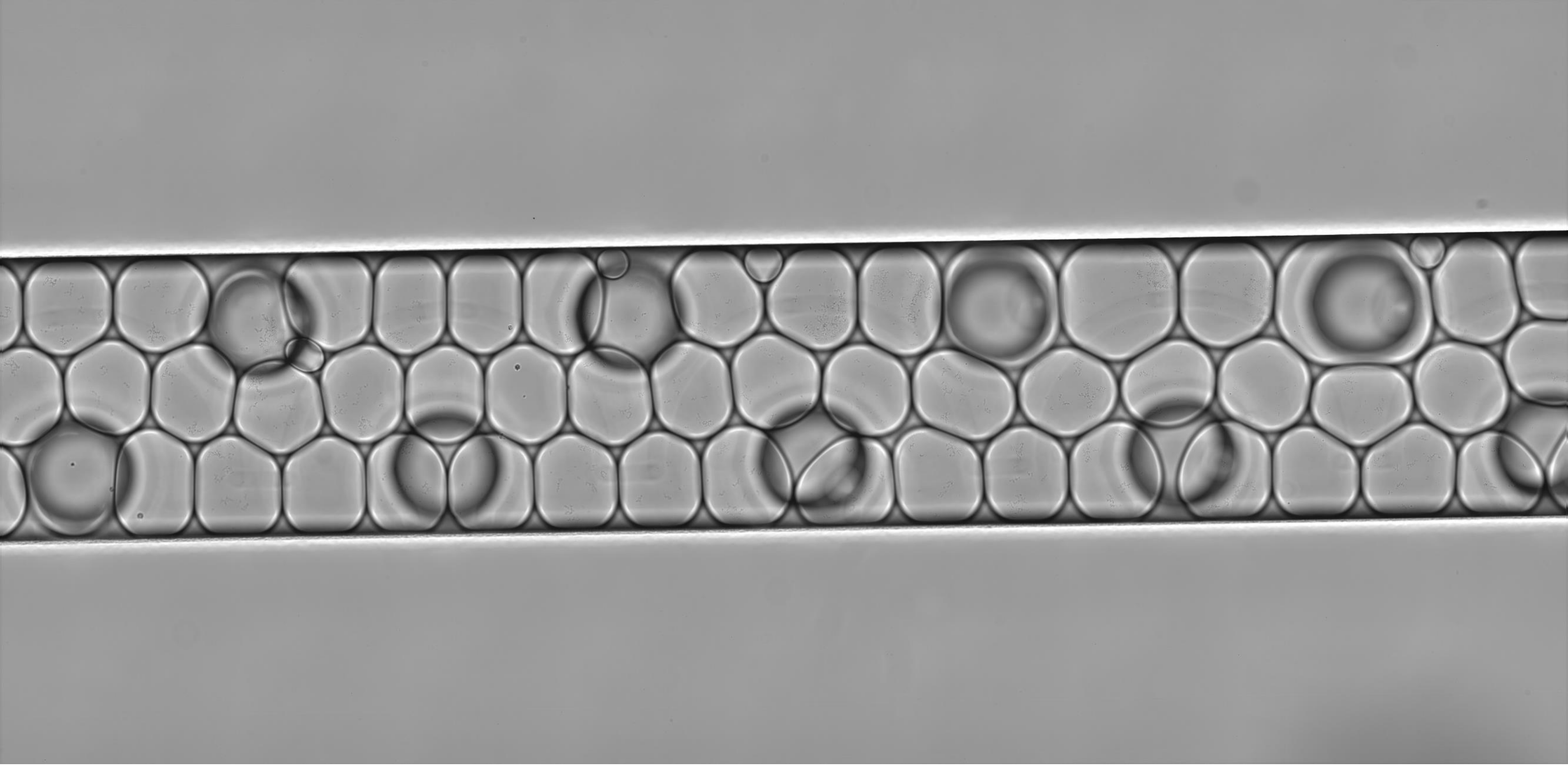

Location: 11 No droplets: 56

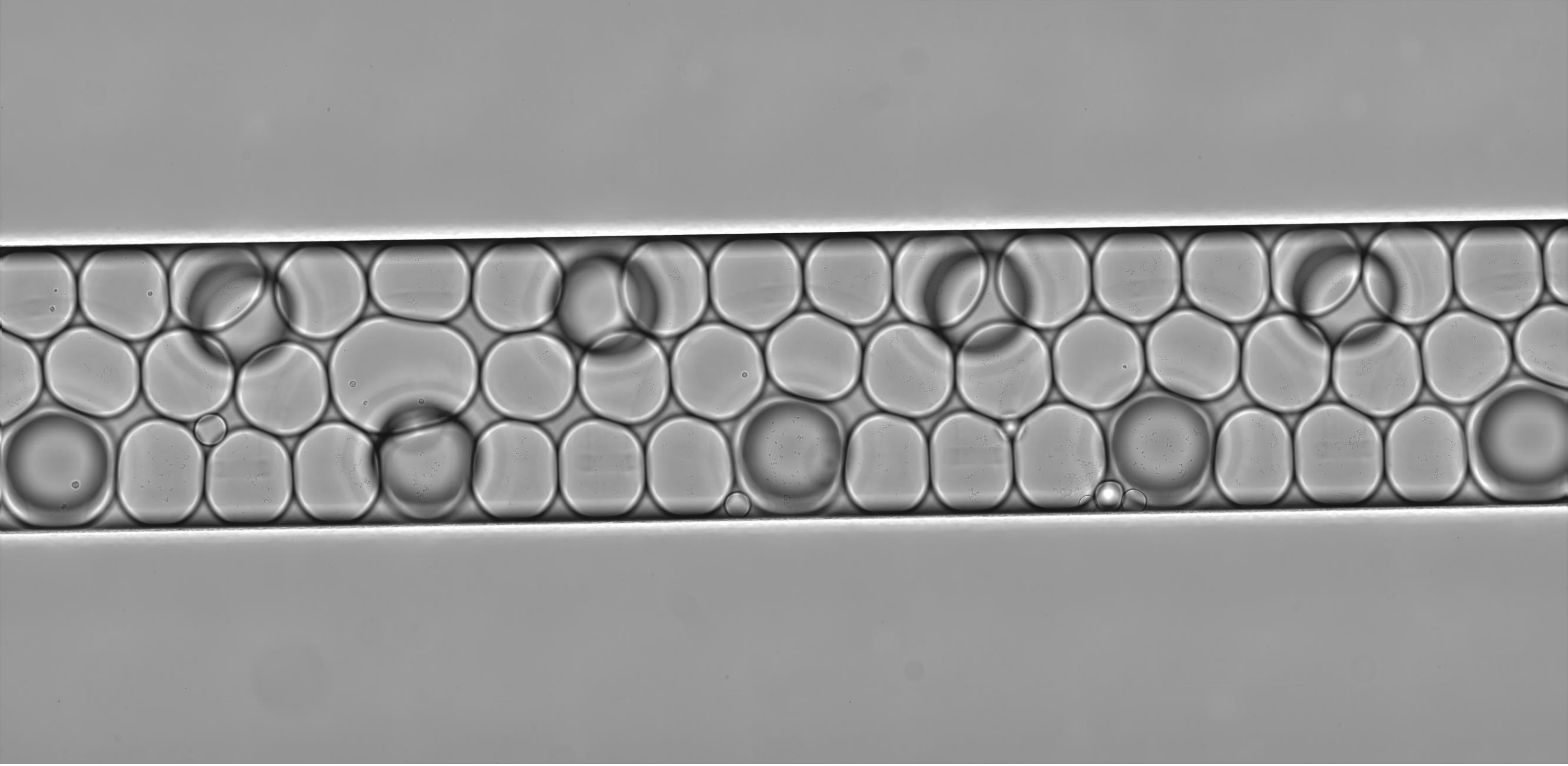

Location: 12 No droplets: 55

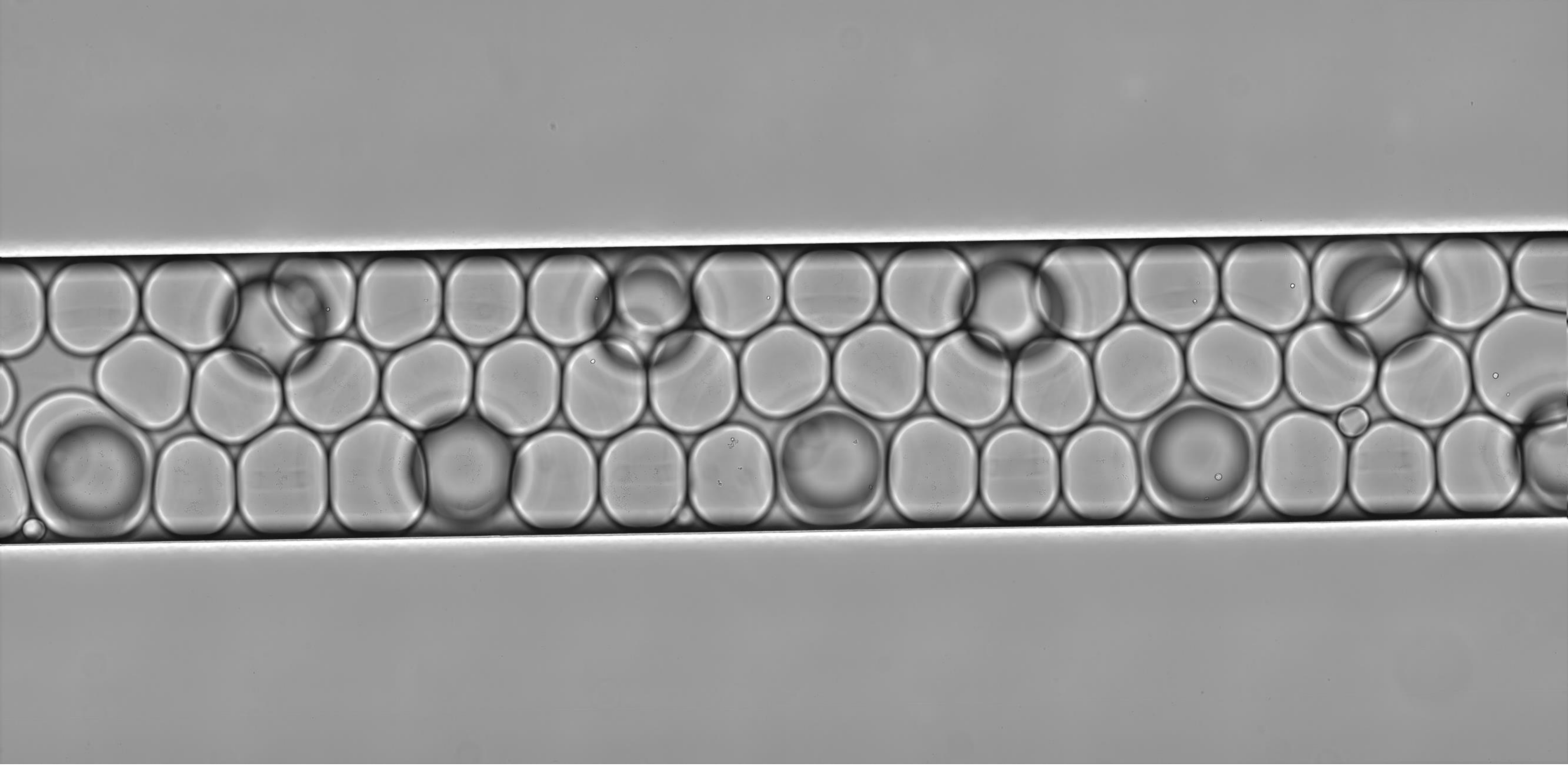

Location: 13 No droplets: 55

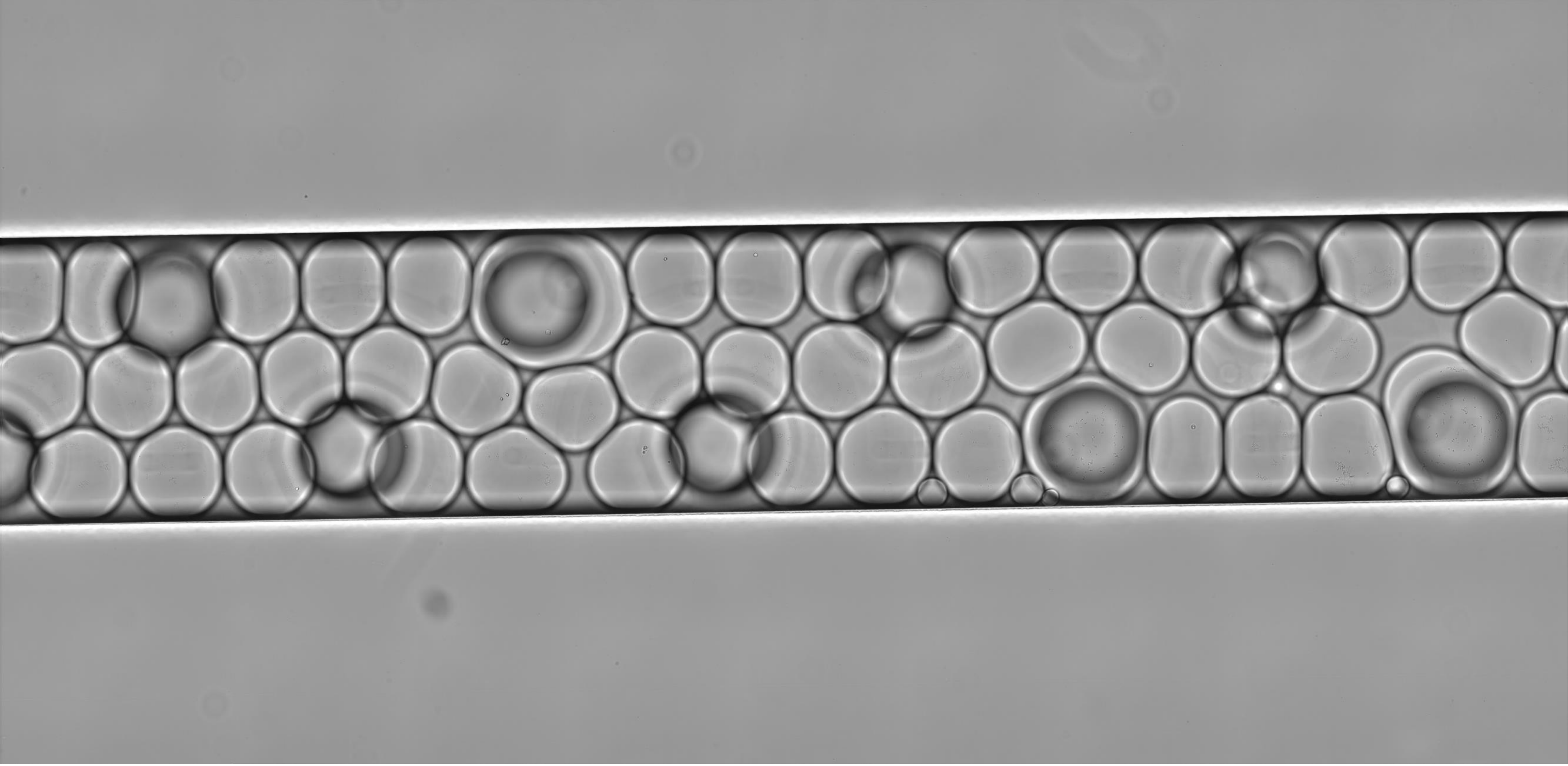

Location: 14 No droplets: 55

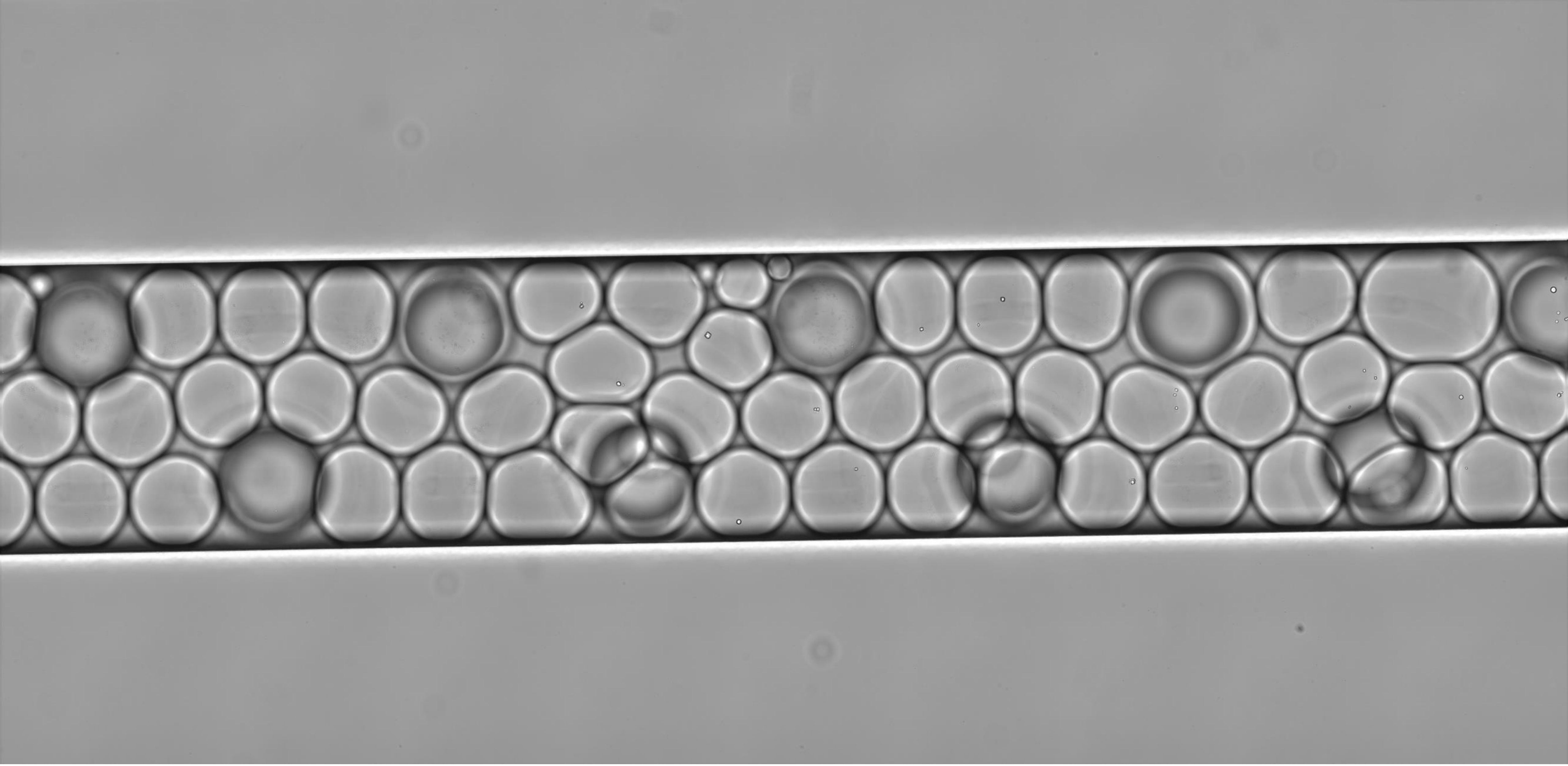

Location: 15 No droplets: 55

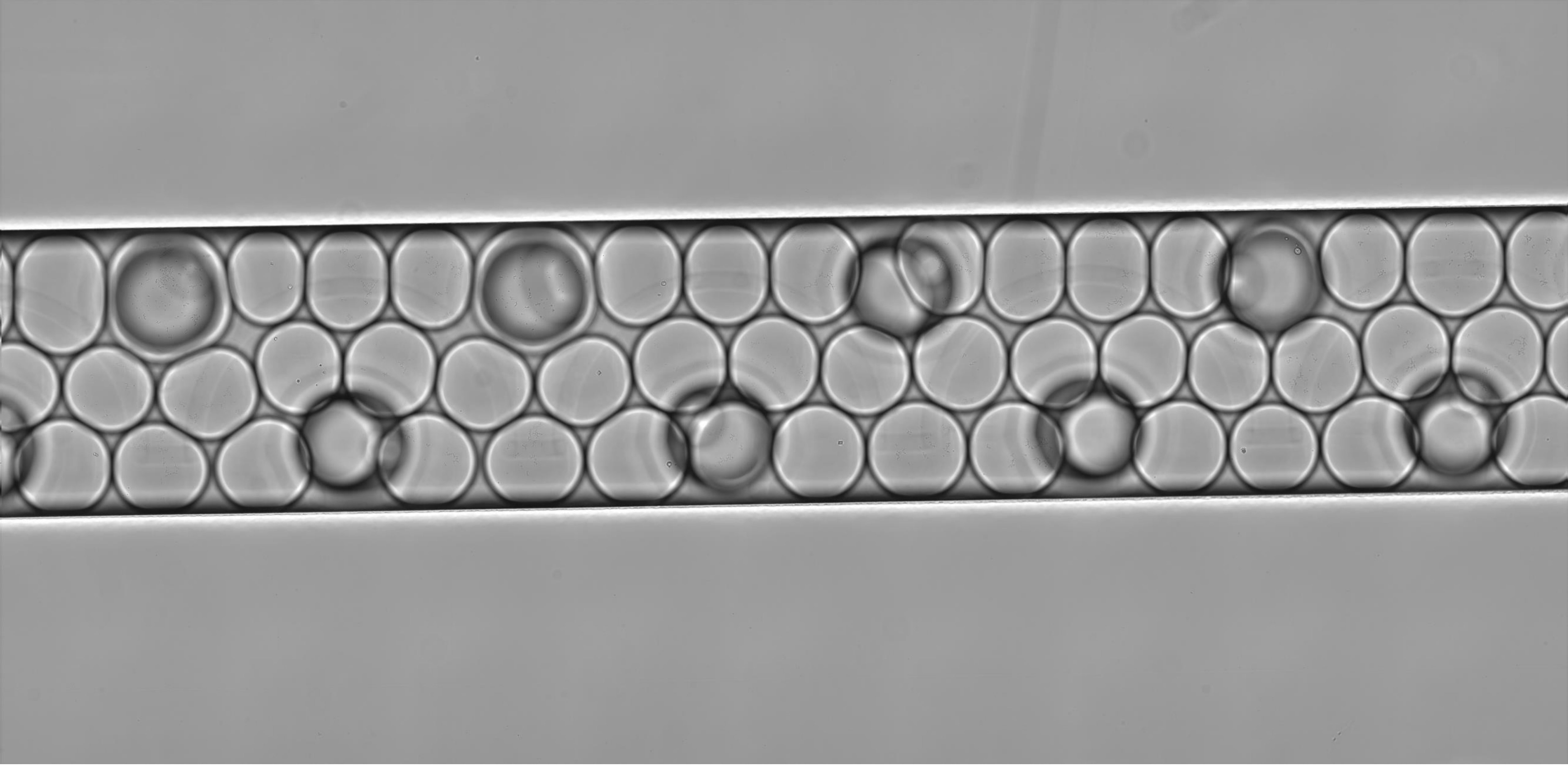

Location: 16 No droplets: 49
