# Supplementary material for: Investigating the metastability of amorphous calcium carbonate by droplet microfluidics experiments using machine learning
Source: Sci Rep. 2025 Jun 20;15:20178. doi: 10.1038/s41598-025-05984-0 (PMC12181231; doi:10.1038/s41598-025-05984-0)
Supplement: Supplementary file 2 — Supplementary Information 2. [file 41598_2025_5984_MOESM2_ESM.pdf]

Supplementary Information 2 for: Investigating the metastability of amorphous calcium carbonate by droplet microfluidics experiments using machine learning

Ryan Santoso[1,*], Lisa Guignon[1,2], Guido Deissmann[1], and Jenna Poonoosamy[1]

[1]Institute of Fusion Energy and Nuclear Waste Management - Nuclear Waste Management (IFN-2), Forschungszentrum Jülich GmbH, 52428 Jülich, Germany
[2]Grenoble INP Ense3, Université Grenoble Alpes, 38000 Grenoble, France
[*]Email: r.santoso@fz-juelich.de

ABSTRACT

This supplementary information provides details on: (1) the construction of each U-Net model within the cascading U-Net method and training samples generation, (2) the implementation of the K-Means clustering method, and (3) the online phase of our proposed method.

1 U-Net

The U-Net is an encoder-decoder model whose architecture consists of contracting and expanding paths[1], shown in Figure 2 in the main text. The model takes an RGB image as input and produces a binary image as output. The contracting path consists of several encoders, where each encoder applies two consecutive 3×3 convolutions (unpadded), followed by batch normalization and Rectified Linear Unit (ReLU) activation. To maintain symmetry, the number of decoders in the expanding path matches the number of encoders[1]. Each decoder applies 2×2 up-sampling, concatenates the result with the corresponding encoder output, and repeats the two 3×3 up-convolutions, each followed by batch normalization and ReLU activation. The final layer consists of a 1×1 convolution followed by a softmax activation. For a detailed mathematical formulation of each operation, readers are encouraged to refer to the literature[2].

The selection of the number of encoders and the number of filters in the first encoder is critical, as it influences the accuracy of the model's predictions. The number of filters doubles with each subsequent encoder after the first, up to the last[1]. Consequently, having more encoders and a higher number of filters in the first encoder increases the number of weights and biases that need to be trained, which raises the risk of overfitting and demands substantial computational resources for training[3,4].

Another important consideration is the selection of hyperparameters, such as learning rate, number of epochs, and batch size. These choices impact how efficiently the model learns, thereby influencing the accuracy of predictions[4,5]. Since selecting optimal hyperparameters is an optimization problem, a balance must be struck between the resources allocated for optimization and the desired level of accuracy[5].

1.1 Generation of training samples

A common approach to generating training samples is to use automatic labeling with software such as ImageJ[6], etc. However, due to the low contrast between the minerals, droplets, and background (see Figure S2.1a), automatic labeling is not feasible in this case. As a result, manual labeling is required to ensure the accurate identification of crystals, droplets, and the background. Consequently, the generation of training samples becomes a labor-intensive process.

Our approach involves labeling in two steps: 1) segmenting the droplets, and 2) cleaning the overlapping droplets and identifying the crystals. For segmenting the droplets (step one), we still rely on ImageJ software[6]. In this step, we focus solely on obtaining sharp droplet boundaries, disregarding the crystals. The procedure is as follows:

1. adjust the contrast between 54 and 224 (due to strong gray color),

2. employ the find-edges function,

3. setting the threshold between 0 and 32,
4. apply Gaussian filter with a standard deviation of 5.

The values for contrast, threshold, and the Gaussian filter's standard deviation are case-dependent and can be tuned for different problems to achieve sharp droplet boundaries. An exemplary result of this first step is shown in Figure S2.1b.

As seen in Figure S2.1b, traces of overlapping droplets remain. It is crucial to remove these overlapping droplets, as they affect the accuracy of the U-Net's predictions. Therefore, in the second step, we use GIMP software[7] to manually remove the black contours representing the overlapping droplets while simultaneously identifying crystals. The steps are as follows:

1. manually erase the boundaries of the overlapping droplets,
2. import the original image as a new layer,
3. superimpose the original image onto the segmented droplet image,
4. trace the crystals and mark the spherical shapes in red and the rhombohedral shapes in green on the superimposed image (the nature of each phase and their typical shapes are identified through Raman spectroscopy, presented in Figure 1e in the main text),
5. remove the original image layer,
6. fill all interstitial areas around the droplets and the background with black.

An exemplary result of this second step is shown in Figure S2.1c.

After applying the procedures above, the original image now has a size of $7975 \times 16381 \times 3$ pixels, and the corresponding label has a size of $7975 \times 16381 \times 1$ pixels. Since the input and output image sizes significantly impact the dimensions of the U-Net model, training with these large images would require a substantial amount of computational resources, making it extremely expensive. Therefore, resizing both the input and output images is essential to enable efficient U-Net model training. The challenge lies in selecting the optimal resizing dimensions.

In our case, we choose 512×512 size as it will not lead to a large and computationally expensive U-Net model. However, we do not directly resize the 7975×16381 image to a 512×512 image since we will loose several mineral features or reduce the pixel count for the minerals, making them harder to be segmented by the U-Net. Instead, we first cut the images and labels into two, then, resize the cut images and labels to 512×512 size, see Figure S2.1d.

Since the U-Net is a data-driven approach, a relatively large number of training samples is required to ensure the model's predictive accuracy[3,4,8]. It is also essential to ensure sufficient variability within the training samples. This variability is achieved through manual labeling of the original images. However, increasing variability through manual labeling alone requires significant labor resources, making it prohibitive to generate an adequate number of training samples. To address this, we apply data augmentation to increase the number of training samples[3,4,9]. The augmentation process randomly applies translation and rotation to the manually labeled samples[4,9]. We use an 80:20 ratio for splitting the samples into training and validation samples.

1.2 Offline phase

The offline phase refers to the process of constructing a machine learning model and optimizing its hyperparameters. In our case, during this phase, we need to train and optimize three U-Net models. Since the second and third U-Net models depend on the results of the first, our strategy is to train the first U-Net model, followed by parallel training of the second and third models. To ensure good predictability, we employ a two-loop approach when training each U-Net model:

1. **The inner loop**. This loop focuses on optimizing the U-Net's weights and biases by minimizing the binary cross-entropy loss between the model's predictions and the ground truth on the training samples:

$$\min_{\mathbf{W},\,\mathbf{b}} L_{\log,\mathrm{tr}} = -\frac{1}{N_{\mathrm{tr}}} \sum_{i=1}^{N_{\mathrm{tr}}} \sum_{k=1}^{K} y_{i,k} \log \Pr(y_{i,k} = 1)_{i,k} \tag{S2.1}$$

where $\mathbf{W}$ is the weights of a U-Net model, $\mathbf{b}$ is the biases of a U-Net model, N_{tr} denotes the number of training samples, K denotes the image dimension, e.g. if our image size is 512×512, $K = 262,144$, y is the value of the true label (in this case, either 0 or 1), and Pr denotes the predicted probability of having $y_{i,k} = 1$. The subscription log denotes logarithmic form, tr denotes training samples, i denotes the enumeration for the training samples, and k denotes the enumeration for image dimension. We use the Keras[10] and TensorFlow Python library[11] to construct the U-Net model and apply gradient descent with the Adam optimizer to solve Equation S2.1.

2. **The outer loop**. This loop is used to optimize the U-Net's hyperparameters by minimizing the binary cross-entropy loss between predictions and ground truth on the validation samples:

$$\min_{\theta} L_{\log,\text{va}} = -\frac{1}{N_{\text{va}}} \sum_{i=1}^{N_{\text{va}}} \sum_{k=1}^{K} y_{i,k} \log \Pr(y_{i,k} = 1)_{i,k} \tag{S2.2}$$

where θ denotes the hyperparameters of a U-Net model. The subscription va denotes validation samples. To solve Equation S2.2, we use a random search approach[2,5] and select the model with the lowest binary cross-entropy loss as the optimal model.

For the first U-Net model, we treat both mineral and droplet pixels as white, and we use an automatic augmentation tool embedded in the optimizer's setup[10,11]. We perform 100 hyperparameter optimization steps. For the second U-Net model, we turn the vaterite pixels white and all other pixels black. Its input is created by overlaying the result of the first U-Net model with the original image. Similarly, for the third U-Net model, we turn calcite pixels white and the rest black, also overlaying the first U-Net model's output with the original image.

2 K-Means clustering method

K-Means clustering is an unsupervised, data-driven machine learning method used to group data into several clusters[12,13]. The algorithm clusters data by minimizing the squared distance between samples and cluster centers[13]:

$$\sum_{k=1}^{K} \min_{\mu_j \in \text{P}} ||y_k - \mu_j||^2 \tag{S2.3}$$

where P is a set of clusters and μ is the cluster center. The subscription j denotes the enumeration for cluster centers. For a detailed mathematical formulation of the K-Means clustering method, readers are encouraged to refer to the literature[13]. In our case, we use the K-Means clustering method to identify cluster members (referred to as labels in Algorithm 2 in the main text) for each pixel in segmented images produced by the first, second, or third U-Net model.

An important consideration is the number of training samples, which in our case is related to the dimensions of the images. With a large number of training samples, the algorithm's performance can significantly degrade, becoming inefficient[14]. Therefore, the choice of image size for segmentation using U-Net has a substantial impact on the performance of K-Means clustering.

2.1 Offline phase

In the offline phase, the K-Means clustering method identifies cluster centers and memberships by solving Lloyd's algorithm[15]. We use 300 iteration steps for this process. As shown in Figure 2 in the main text, we train three K-Means models in parallel since they operate independently of each other.

3 Online phase

The online phase refers to deploying trained machine learning models for prediction. The process during the online phase is as follows:

1. The phase begins by feeding cut RGB snapshots from the droplet microfluidics experiment into the first U-Net model.
2. Once the first U-Net model delivers predictions, the results are overlaid with the corresponding RGB snapshots.
3. These overlaid images are then passed to the second and third U-Net models for the identification of vaterite and calcite, respectively.
4. The results from each U-Net model are processed through the K-Means clustering method to count the number of droplets, and vaterite and calcite crystals.
5. The outputs are further stacked and passed into Algorithm 2 to count the number of droplets containing the ACC phase.
6. The cut predictions are merged to form the final result.

References

1. Ronneberger, O., Fischer, P. & Brox, T. U-net: Convolutional networks for biomedical image segmentation. In *Medical image computing and computer-assisted intervention–MICCAI 2015: 18th international conference, Munich, Germany, October 5-9, 2015, proceedings, Part III 18*, 234–241 (Springer, 2015).
2. Bishop, C. M. & Nasrabadi, N. M. *Pattern recognition and machine learning*, vol. 4 (Springer, 2006).
3. Micallef, N., Seychell, D. & Bajada, C. J. Exploring the U-net++ model for automatic brain tumor segmentation. *IEEE Access* **9**, 125523–125539 (2021).
4. Santoso, R., He, X. & Hoteit, H. Application of machine-learning to construct simulation models from high-resolution fractured formation. In *Abu Dhabi International Petroleum Exhibition and Conference*, D021S060R004 (SPE, 2019).
5. Yang, L. & Shami, A. On hyperparameter optimization of machine learning algorithms: Theory and practice. *Neurocomputing* **415**, 295–316 (2020).
6. Schneider, C. A., Rasband, W. S. & Eliceiri, K. W. NIH Image to ImageJ: 25 years of image analysis. *Nat. methods* **9**, 671–675 (2012).
7. Whitt, P. *Beginning photo retouching and restoration using GIMP* (Apress, 2014).
8. Swischuk, R., Mainini, L., Peherstorfer, B. & Willcox, K. Projection-based model reduction: Formulations for physics-based machine learning. *Comput. & Fluids* **179**, 704–717 (2019).
9. Shorten, C. & Khoshgoftaar, T. M. A survey on image data augmentation for deep learning. *J. Big Data* **6**, 1–48 (2019).
10. Chollet, F. *et al.* Keras. https://github.com/fchollet/keras (2015).
11. Abadi, M. *et al.* TensorFlow: Large-scale machine learning on heterogeneous systems (2015). Software available from tensorflow.org.
12. Ikotun, A. M., Ezugwu, A. E., Abualigah, L., Abuhaija, B. & Heming, J. K-means clustering algorithms: A comprehensive review, variants analysis, and advances in the era of big data. *Inf. Sci.* **622**, 178–210 (2023).
13. Blömer, J., Lammersen, C., Schmidt, M. & Sohler, C. Theoretical analysis of the K-means algorithm–a survey. *Algorithm Eng. Sel. Results Surv.* 81–116 (2016).
14. Arthur, D. & Vassilvitskii, S. How slow is the K-means method? In *Proceedings of the twenty-second annual symposium on Computational geometry*, 144–153 (2006).
15. Pedregosa, F. *et al.* Scikit-learn: Machine learning in Python. *J. Mach. Learn. Res.* **12**, 2825–2830 (2011).

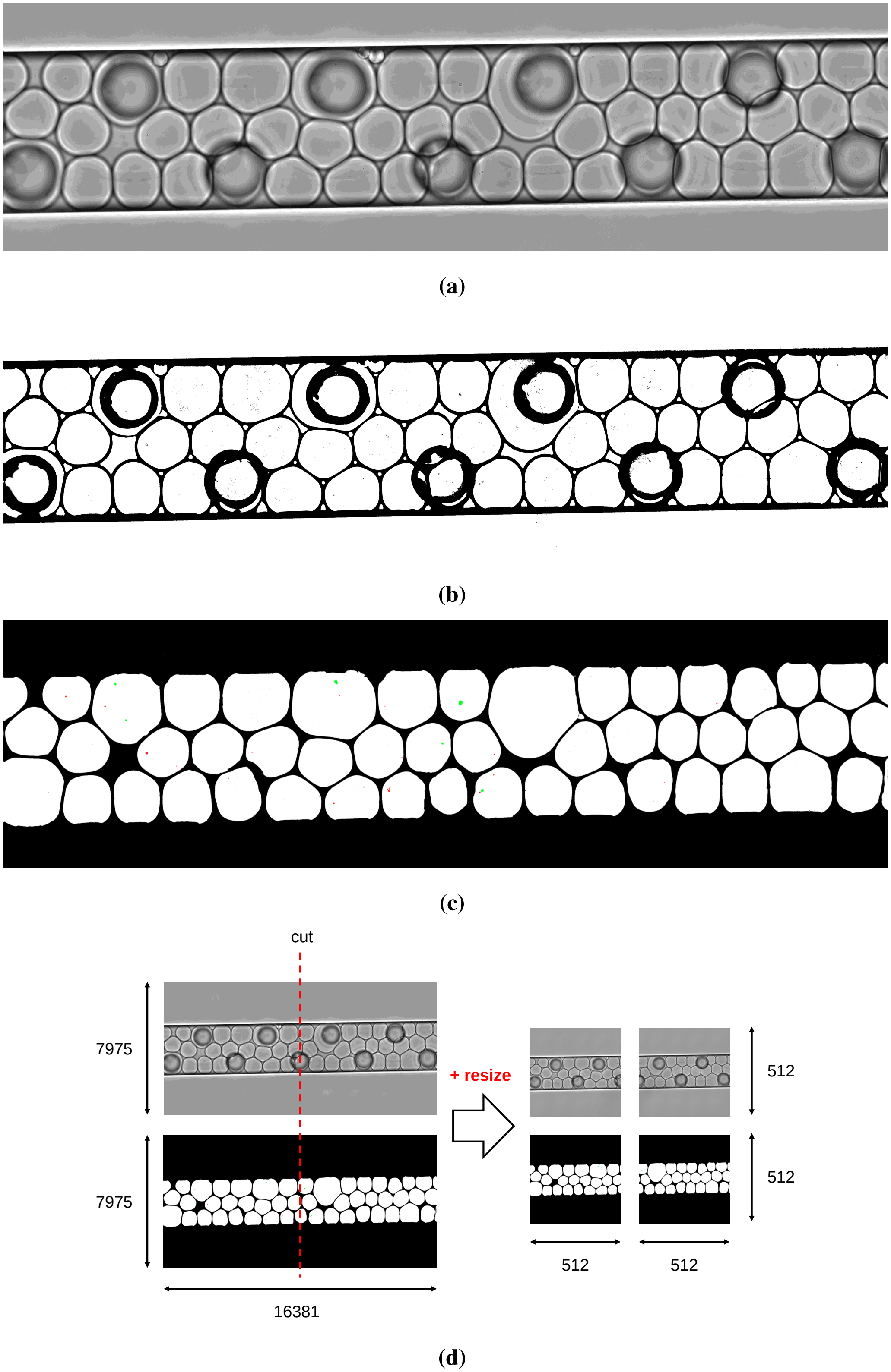

Figure S2.1. Exemplary results obtained during the training samples generation process: (a) the original image, (b) the result after applying step one, (c) the result after applying step two, and (d) the final images and labels for the U-Net training.
