# Supplementary material for: Investigating the metastability of amorphous calcium carbonate by droplet microfluidics experiments using machine learning
Source: Sci Rep. 2025 Jun 20;15:20178. doi: 10.1038/s41598-025-05984-0 (PMC12181231; doi:10.1038/s41598-025-05984-0)
Supplement: Supplementary file 3 — Supplementary Information 3. [file 41598_2025_5984_MOESM3_ESM.pdf]

Supplementary Information 3 for:

Investigating the metastability of amorphous calcium carbonate by droplet microfluidics experiments using machine learning

Ryan Santoso[1], Lisa Guignon[1,2], Guido Deissmann[1], and Jenna Poonoosamy[1]

[1]Institute of Fusion Energy and Nuclear Waste Management – Nuclear Waste Management (IFN-2), Forschungszentrum Jülich GmbH, 52428 Jülich, Germany

[2]Grenoble INP Ense3, Université Grenoble Alpes, 38000 Grenoble, France

ABSTRACT

This supplementary information provides snapshots after 24 hours showing that most droplets are still containing the ACC phase.

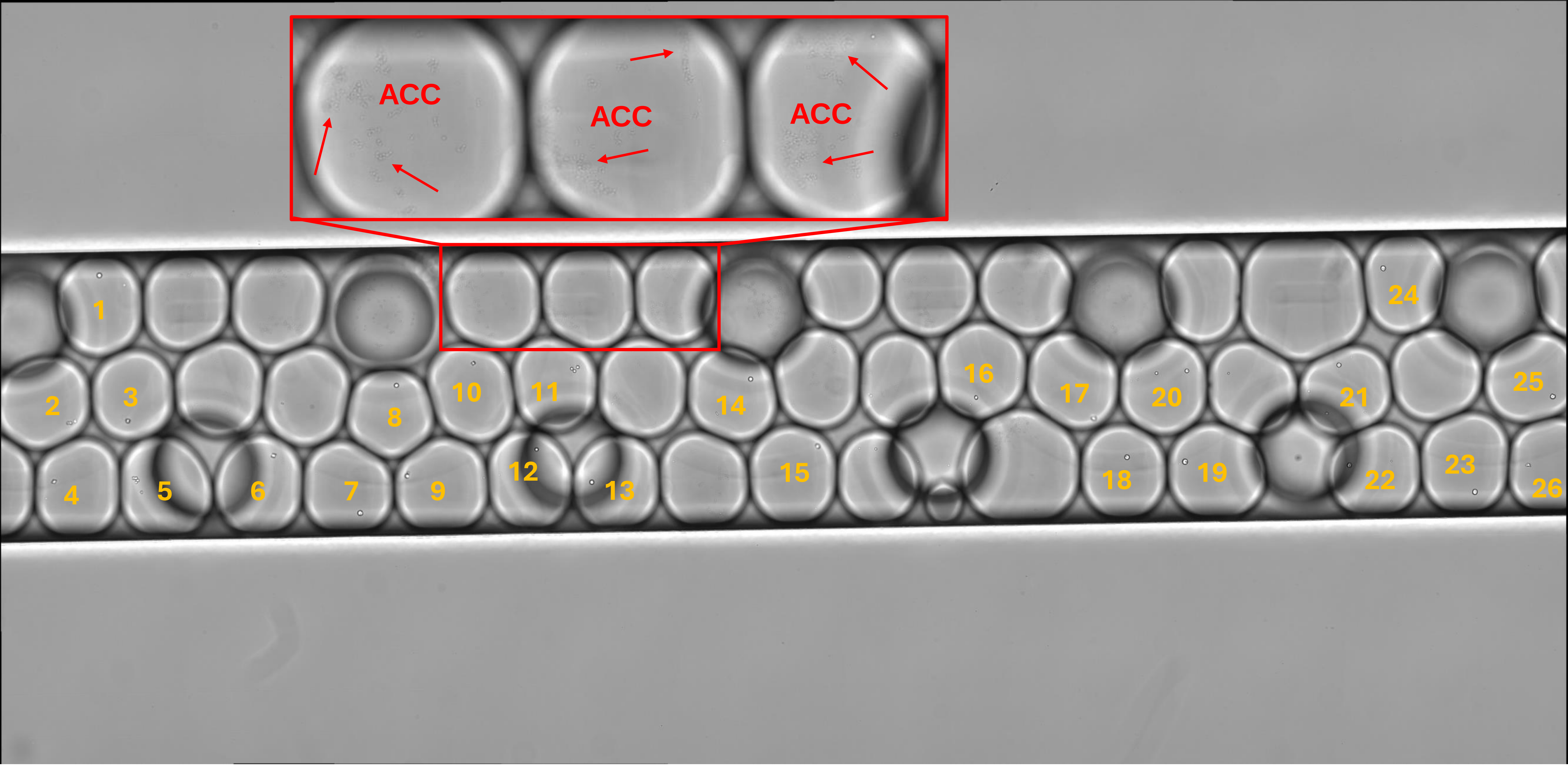

Location: 4 No droplets: 53 No droplets with ACC: 53 – 26 = 27

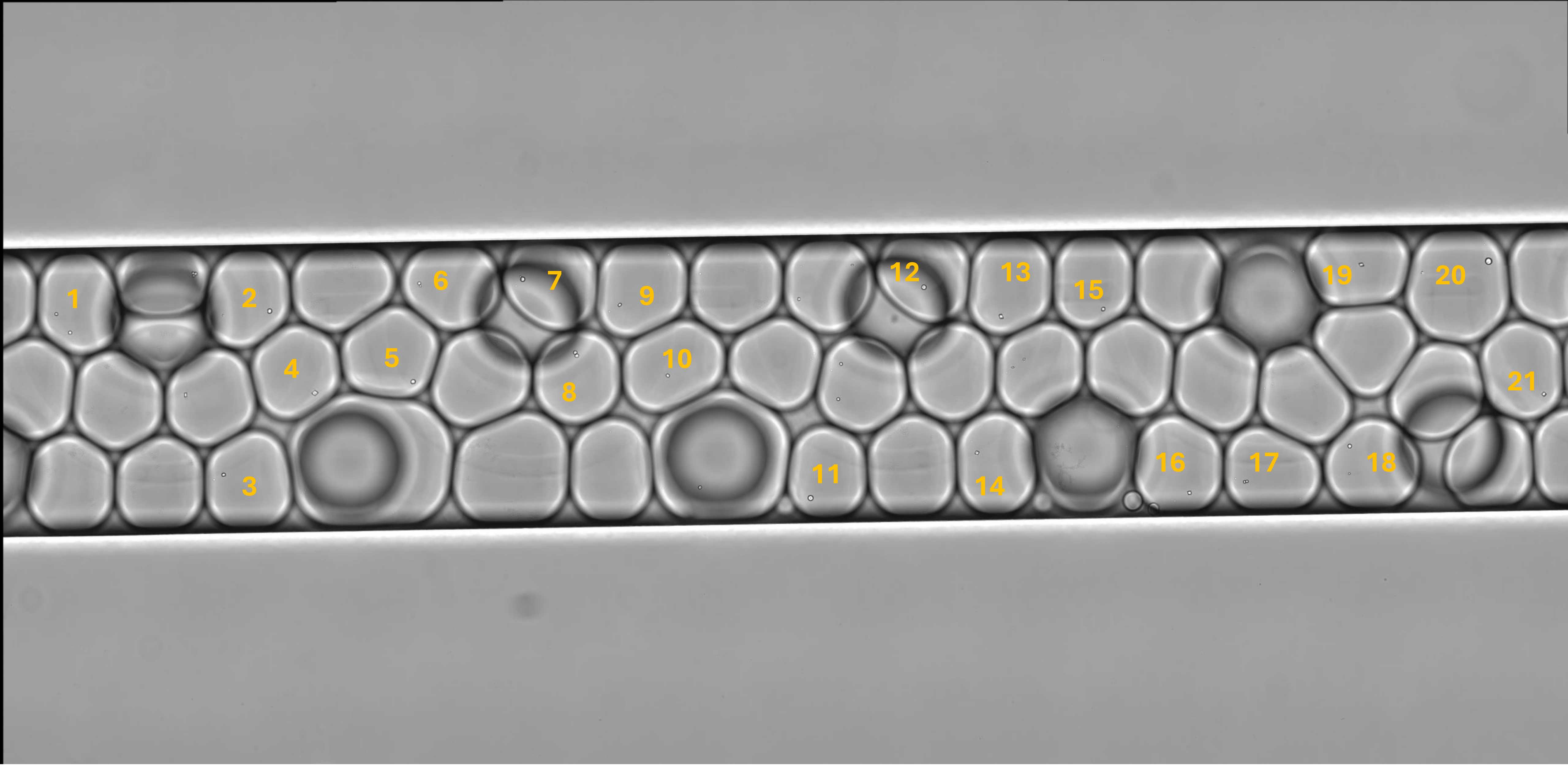

Location: 6

No droplets: 57

No droplets with ACC: 57 – 21 = 36

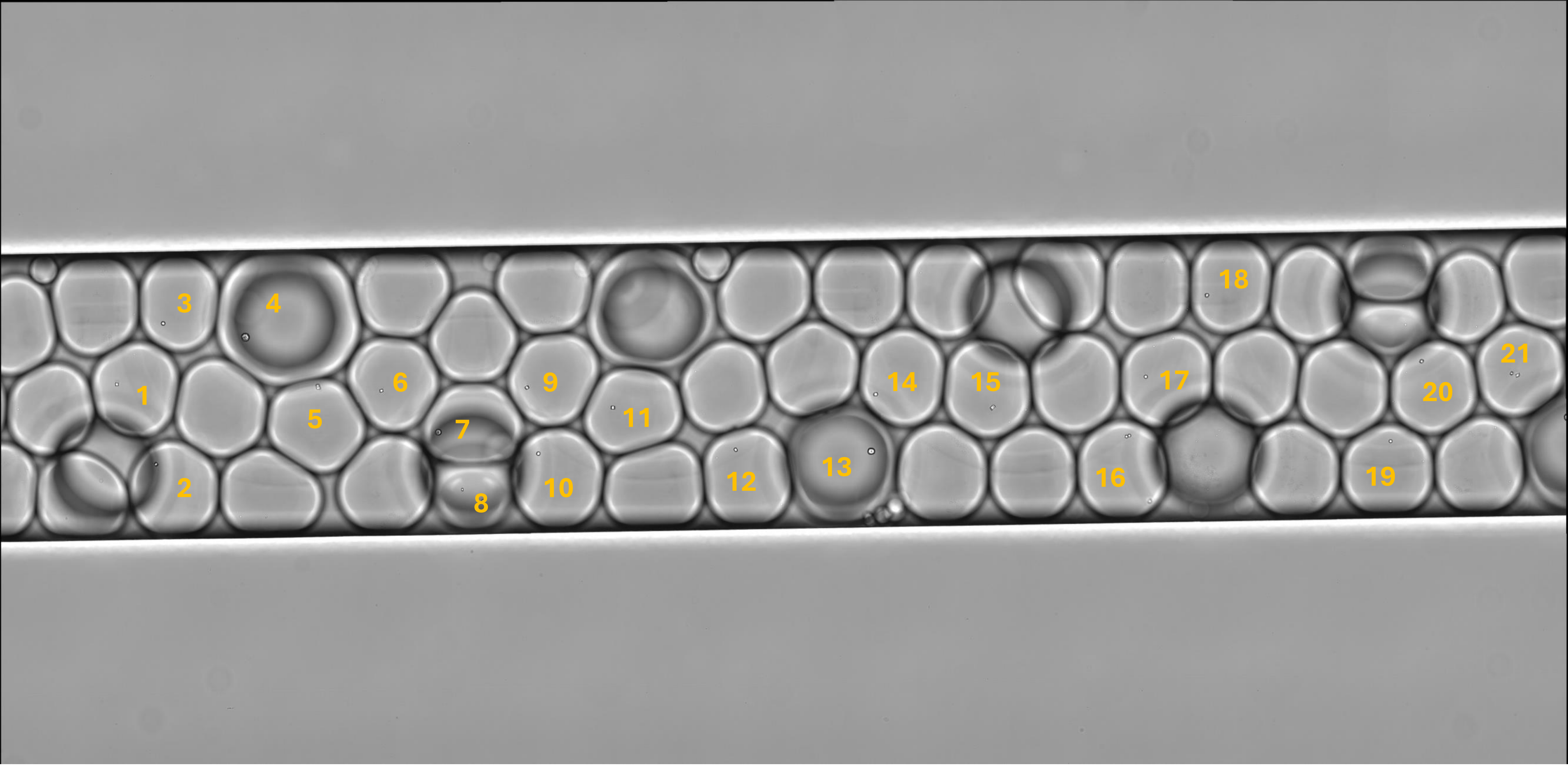

Location: 7

No droplets: 59

No droplets with ACC: 59 – 21 = 38

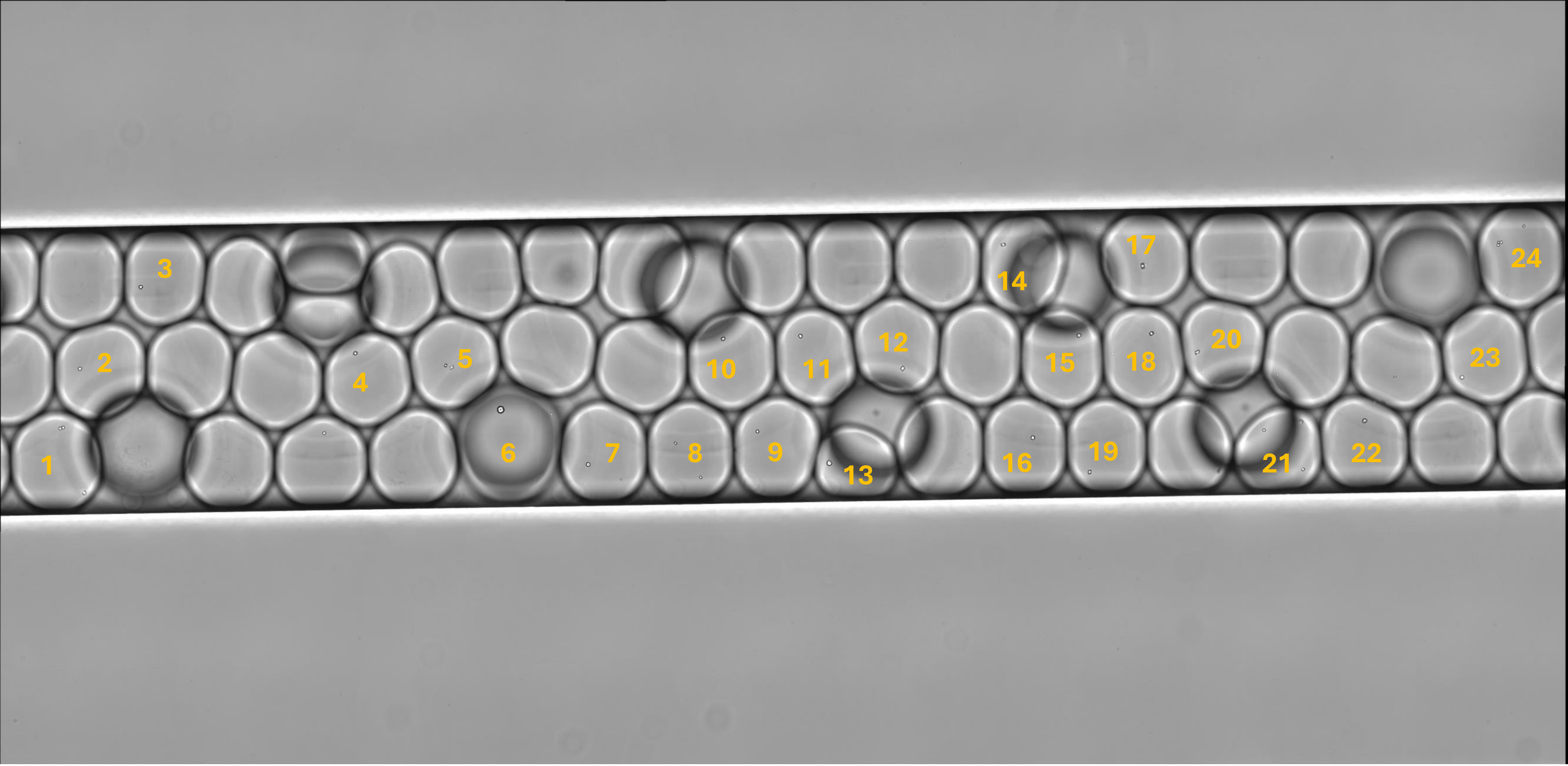

Location: 8 No droplets: 57 No droplets with ACC: 57 – 24 = 33

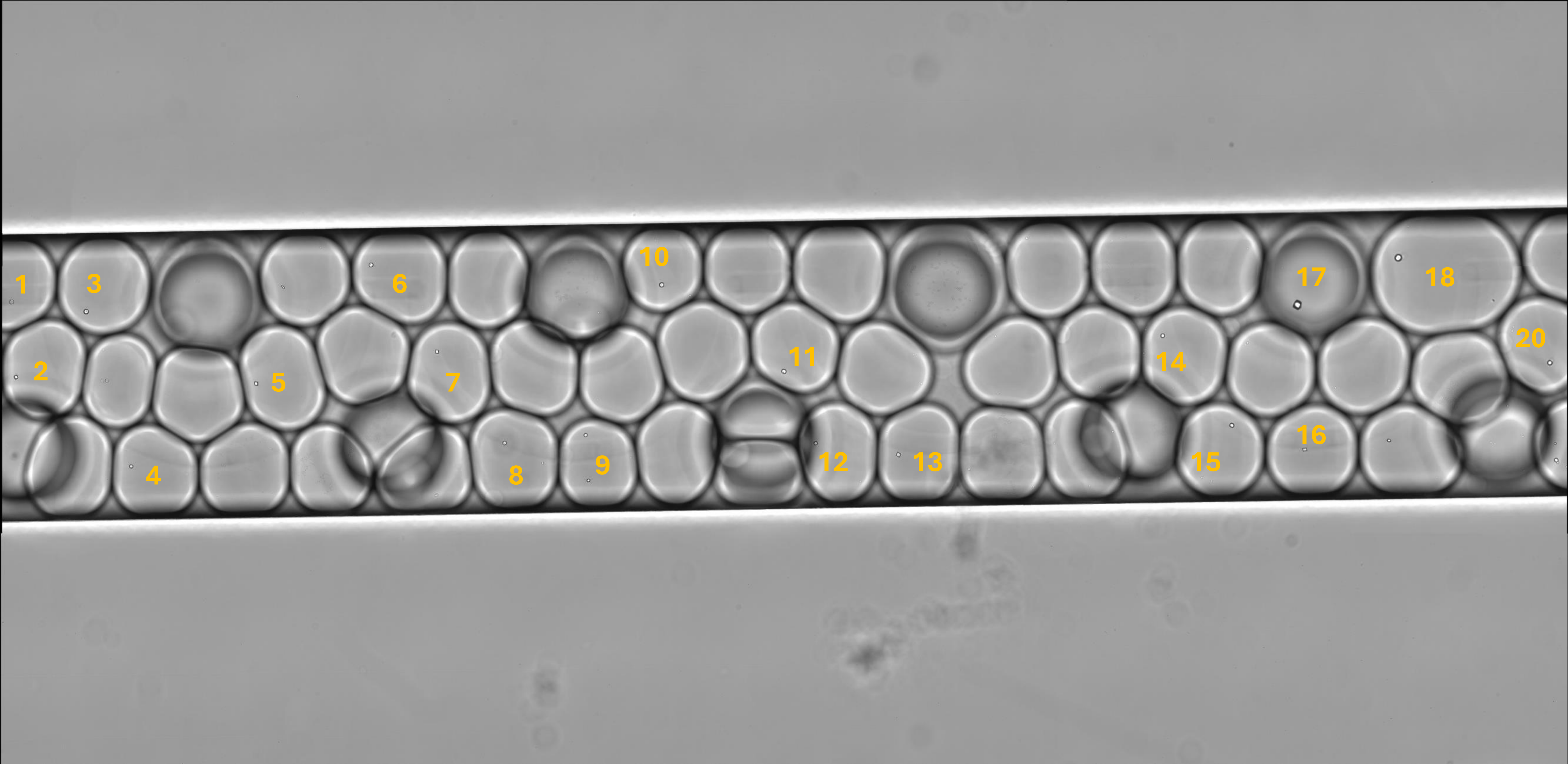

Location: 9 No droplets: 53 No droplets with ACC: 53 – 20 = 33

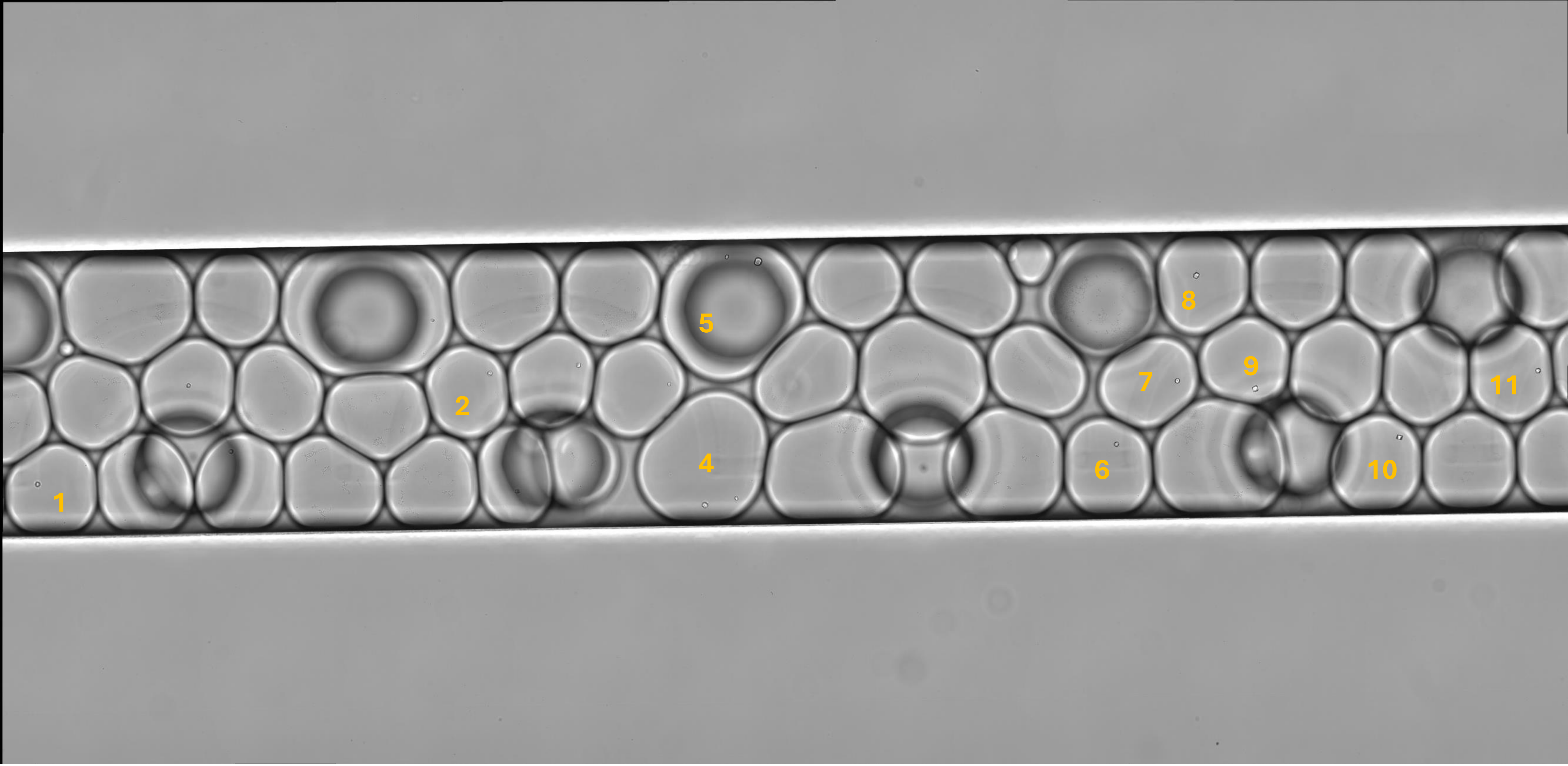

Location: 10

No droplets: 49

No droplets with ACC: 49 – 11 = 38

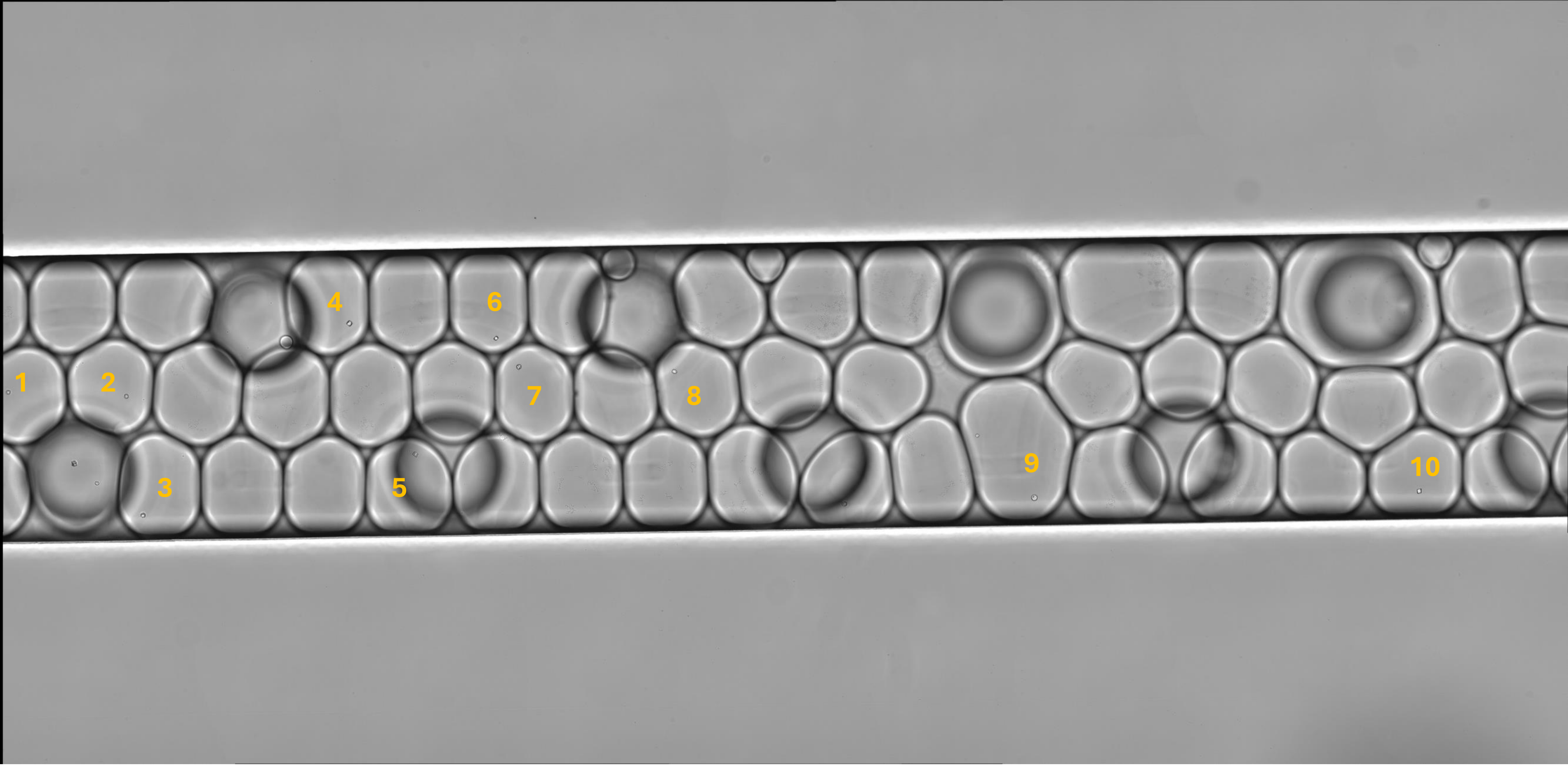

Location: 11 No droplets: 56 No droplets with ACC: 56 – 10 = 46

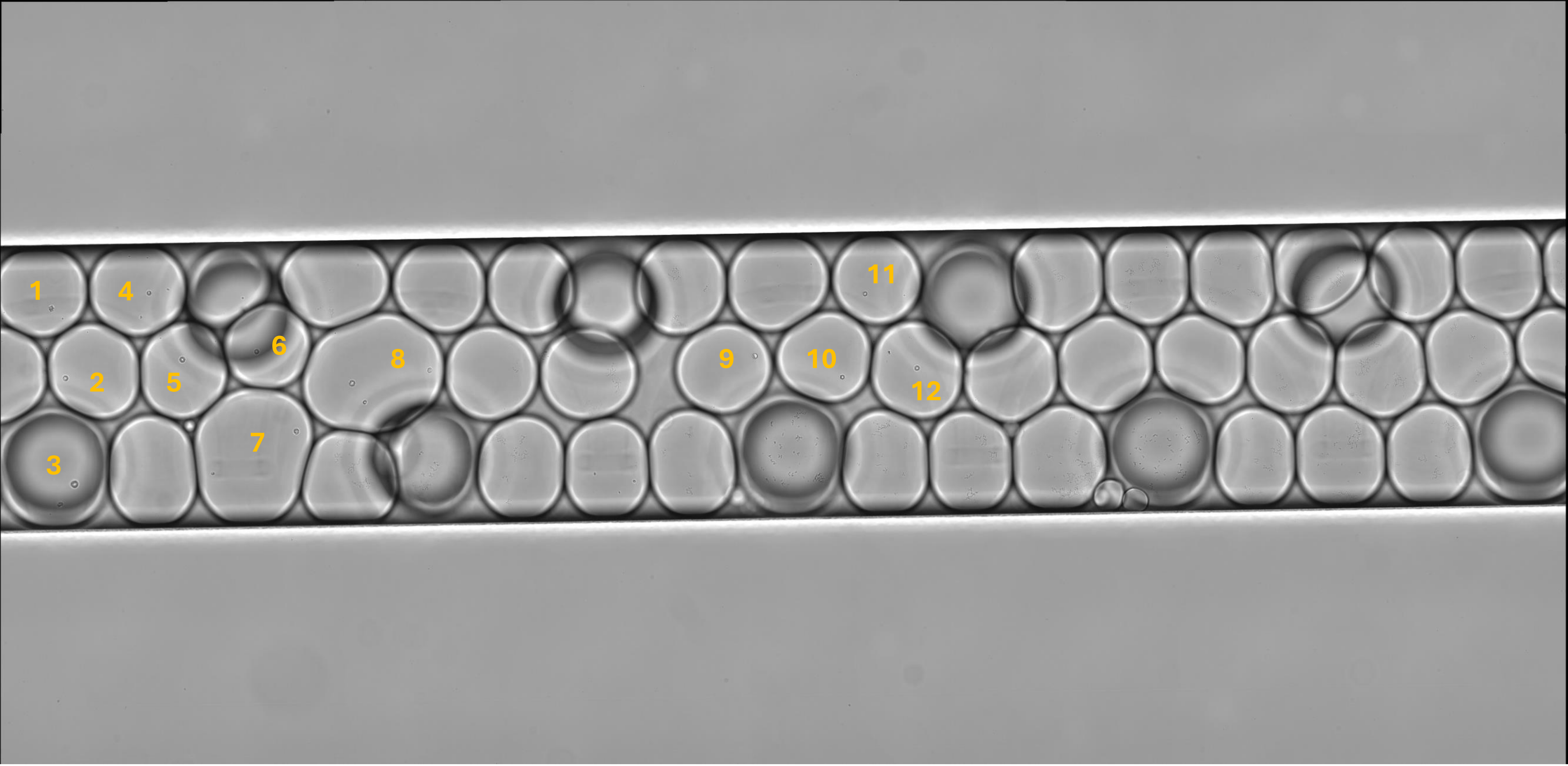

Location: 12

No droplets: 55

No droplets with ACC: 55 – 12 = 43

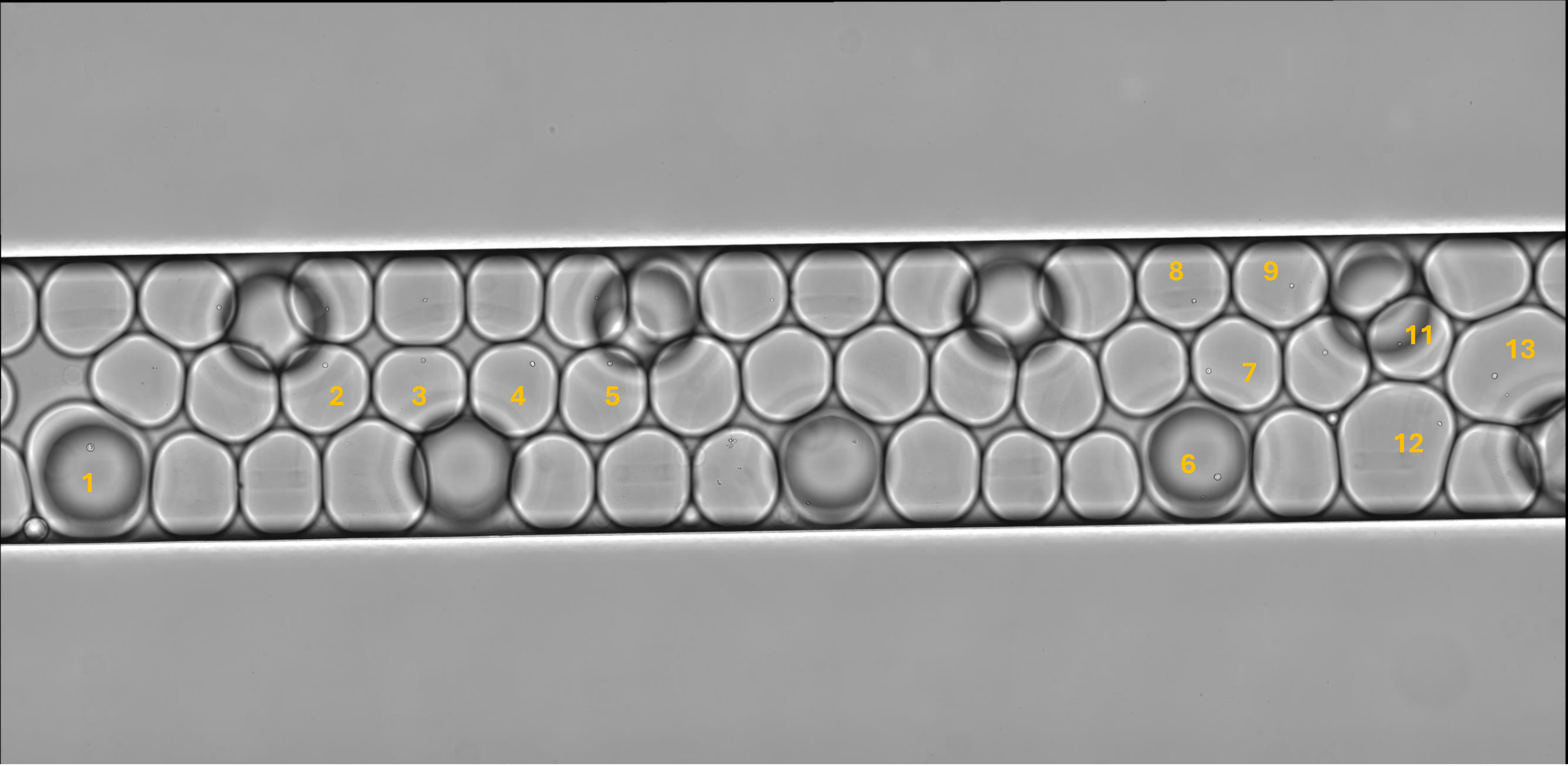

Location: 13 No droplets: 55 No droplets with ACC: 55 – 13 = 42

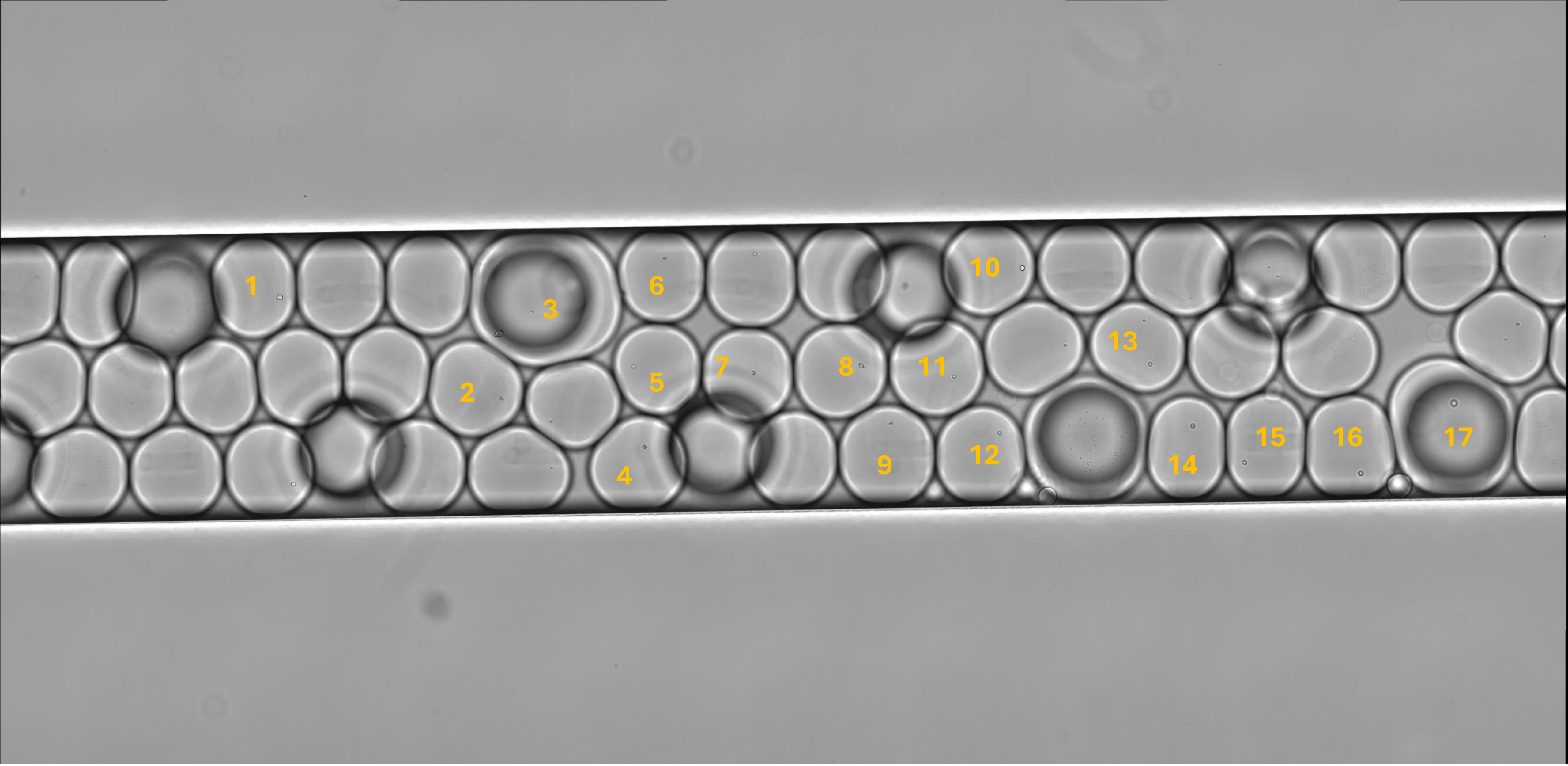

Location: 14 No droplets: 55 No droplets with ACC: 55 – 17 = 38

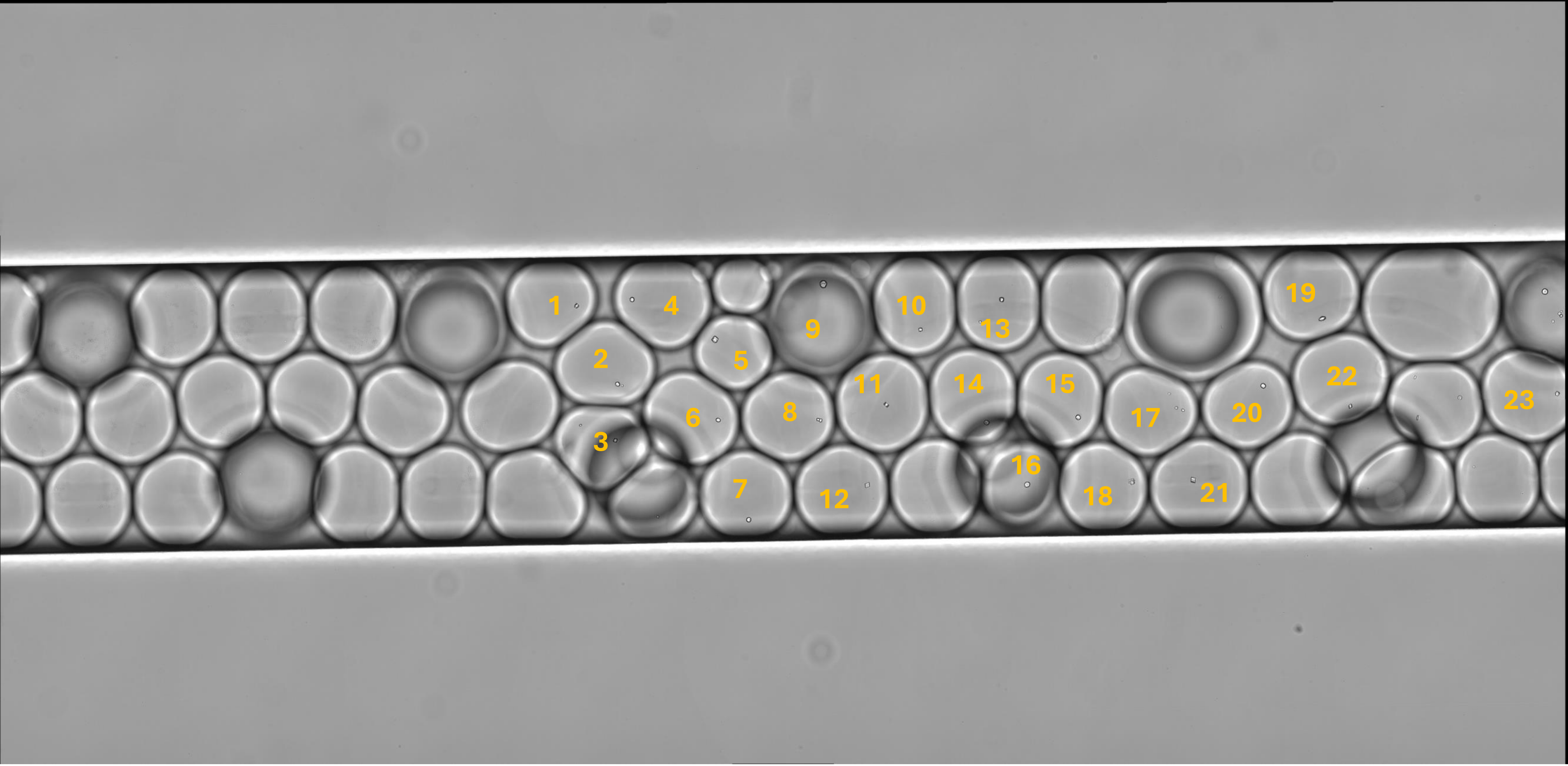

Location: 15 No droplets: 55 No droplets with ACC: 55 – 23 = 32

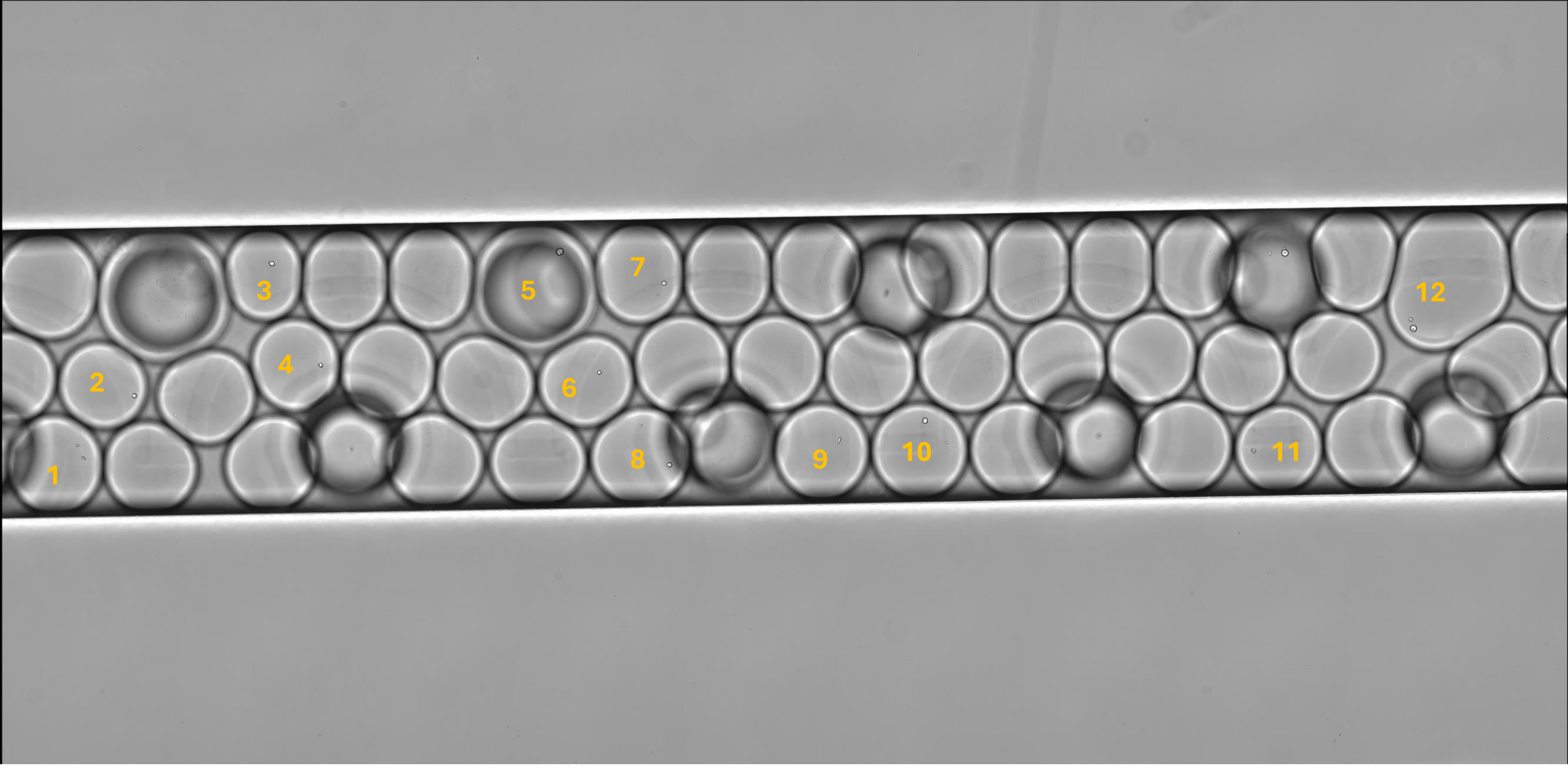

Location: 16 No droplets: 49 No droplets with ACC: 49 – 12 = 37
