# Supplementary material for: Investigating the metastability of amorphous calcium carbonate by droplet microfluidics experiments using machine learning
Source: Sci Rep. 2025 Jun 20;15:20178. doi: 10.1038/s41598-025-05984-0 (PMC12181231; doi:10.1038/s41598-025-05984-0)
Supplement: Supplementary file 4 — Supplementary Information 4. [file 41598_2025_5984_MOESM4_ESM.pdf]

Supplementary Information 4 for: Investigating the metastability of amorphous calcium carbonate by droplet microfluidics experiments using machine learning

Ryan Santoso[1,*], Lisa Guignon[1,2], Guido Deissmann[1], and Jenna Poonoosamy[1]

[1]Institute of Fusion Energy and Nuclear Waste Management - Nuclear Waste Management (IFN-2), Forschungszentrum Jülich GmbH, 52428 Jülich, Germany
[2]Grenoble INP Ense3, Université Grenoble Alpes, 38000 Grenoble, France
[*]Email: r.santoso@fz-juelich.de

ABSTRACT

This supplementary information provides details on the construction of a DeepLabV3+ model and its performance.

1 DeepLabV3+

We apply the DeepLabV3+ model[1,2] to our training samples, as described in section 1.1 in Supplementary Information 2, for comparison with the cascading U-Net method. The DeepLabV3+ model is an extension of DeepLabV3 and shares a similar architecture with the U-Net model[1]. It employs Atrous Spatial Pyramid Pooling (ASPP) to achieve accurate multi-label segmentation, which the U-Net model is less equipped to handle[1]. Additionally, it incorporates a pre-trained model, such as the ResNet[3] or Xception[4] model, as the backbone[1]. For a more detailed discussion of the model, readers are encouraged to check the literature[1].

For training the DeepLabV3+ model, we employ the same two-loop approach described in section 1.2 in Supplementary Information 2. Instead of minimizing the binary cross-entropy loss, the model minimizes the categorical cross-entropy loss:

$$L_{\log} = -\frac{1}{N}\sum_{i=1}^{N}\sum_{k=1}^{K}\sum_{c=1}^{C} y_{i,c,k} \log \Pr(y_{i,c,k})_{i,c,k} \tag{S4.1}$$

where N is the number of samples, can be either training or validation samples, K the image dimension, and C is the set of labels. The subscription log denotes logarithmic form, c denotes the enumeration of labels, i denotes the enumeration for the samples, and k denotes the enumeration for image dimension. For hyperparameter optimization, we tune the learning rate, number of epochs, and batch size. The architecture follows the construction outlined in the literature[1]. We again use 100 optimization steps. The augmentations are embedded within the optimizers, following the Keras[5]/Tensorflow setup[6]. This implementation is within our **deeplab** Python library in our Github repository `https://github.com/FZJ-RT/deeplab.git`.

2 Results

As shown in Figure S4.1, the DeepLabV3+ model produces noisy predictions of the droplets boundaries. This noise appears in the tube wall segmentation due to the similar contrast between the tube walls and the droplets boundaries, causing the model to misidentify the tube walls as part of the droplets boundaries. Additionally, the model incorrectly predicts vaterite pixels and fails to identify calcite pixels. This suggests that the model requires more training samples, both through manual labeling and data augmentation, to improve accuracy.

Table S4.1 shows the performance of the DeepLabV3+ model. The offline and online time for this model are comparable to those of the U-Net model in our method. However, with the current number of training samples, the error remains relatively high, indicating the need for more training data.

Table S4.1. Performance of DeepLabV3+ model. For offline phase, we use a GPU in JURECA High-Performance Computing (HPC) infrastructure at Forschungszentrum Jülich, utilizing a GPU-equipped node with 2 × AMD EPYC 7742 processors (2 × 64 CPU cores at 2.25 GHz), 512 GB of RAM, and 4 × NVIDIA A100 GPUs per node. For online phase, we employ a CPU in a Dell Latitude 7440 Laptop equipped with Gen 13th Intel i7-1365U containing 12 CPU cores at 1.8 GHz and 32 GB RAM.

Learning rate	Batch size	Number of epochs	Offline time (minutes)	Offline resources	Categorical cross-entropy error	Online time per image (seconds)
1.0×10^{-5}	8	4,802	18	1 GPU	2.9×10^{6}	0.5

References

1. Chen, L.-C., Zhu, Y., Papandreou, G., Schroff, F. & Adam, H. Encoder-decoder with atrous separable convolution for semantic image segmentation. In *Proceedings of the European conference on computer vision (ECCV)*, 801–818 (2018).
2. Chen, L.-C., Papandreou, G., Schroff, F. & Adam, H. Rethinking atrous convolution for semantic image segmentation. arxiv. *arXiv preprint arXiv:1706.05587* **5**, 1–14 (2017).
3. He, K., Zhang, X., Ren, S. & Sun, J. Deep residual learning for image recognition. In *Proceedings of the IEEE conference on computer vision and pattern recognition*, 770–778 (2016).
4. Chollet, F. Xception: Deep learning with depthwise separable convolutions. In *Proceedings of the IEEE conference on computer vision and pattern recognition*, 1251–1258 (2017).
5. Chollet, F. *et al.* Keras. https://github.com/fchollet/keras (2015).
6. Abadi, M. *et al.* TensorFlow: Large-scale machine learning on heterogeneous systems (2015). Software available from tensorflow.org.

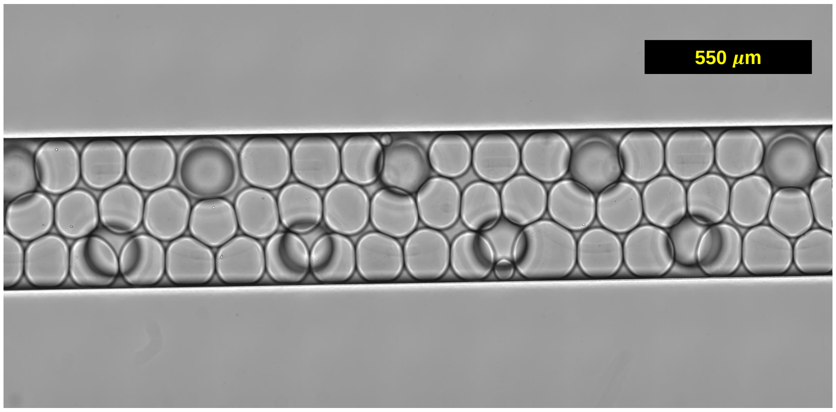

(a)

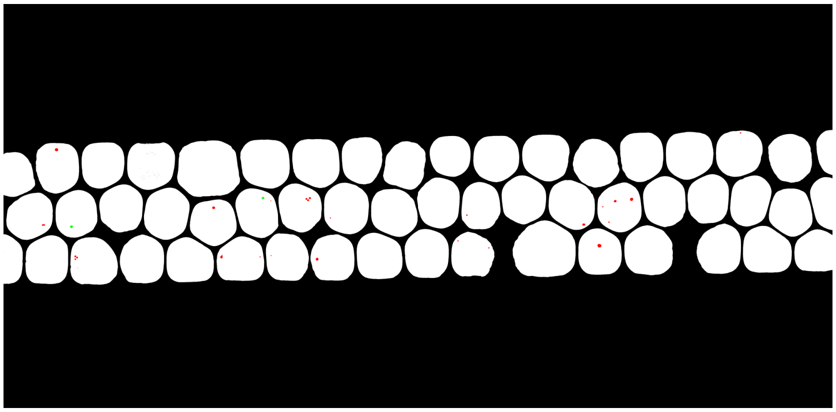

(b)

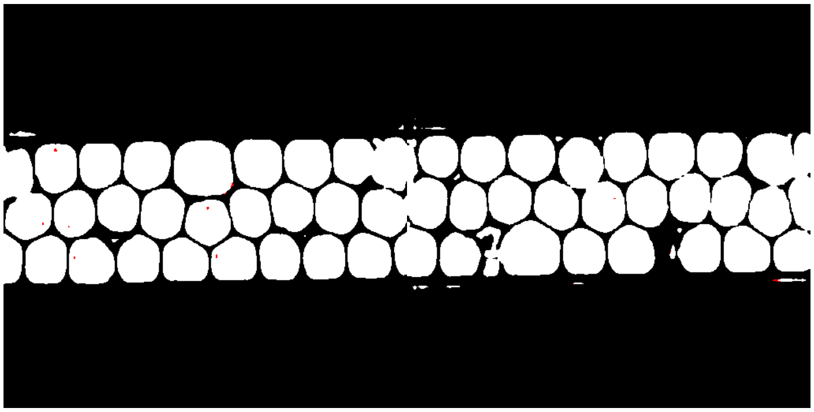

(c)

Figure S4.1. An exemplary segmentation result using our DeepLabV3+ model: (a) the experimental snapshot at time = 22.72 minutes, (b) the ground truth, and (c) the prediction result. The presented images (a) to (c) are combined images after going through cutting process in Figure S2.1 in Supplementary Information 2. The green pixels indicate calcite crystals, red pixels indicate vaterite crystals, and bubbles without both red and green pixels are classified as containing the ACC phase.
